# Synthesis, Spectroscopic Characterization, and Biological Evaluation of a Novel Acyclic Heterocyclic Compound: Anticancer, Antioxidant, Antifungal, and Molecular Docking Studies

**DOI:** 10.3390/ph18101533

**Published:** 2025-10-12

**Authors:** Mohammad Alhilal, Suzan Alhilal, Ilhan Sabancilar, Sobhi M. Gomha, Ahmed A. Elhenawy, Salama A. Ouf

**Affiliations:** 1 Department of Nursing, Faculty of Health Sciences, Mardin Artuklu University, Mardin 47200, Turkey; mohammadalhilal@artuklu.edu.tr; 2 Department of Medical Services and Techniques, Vocational School of Health Services, Mardin Artuklu University, Mardin 47200, Turkey; 3 Department of Medical Services and Techniques, Vocational School of Health Services, Bitlis Eren University, Bitlis 13100, Turkey; isabancilar@beu.edu.tr; 4 Department of Chemistry, Faculty of Science, Islamic University of Madinah, Madinah 42351, Saudi Arabia; 5 Chemistry Department, Faculty of Science, Al-Baha University, Al-Baha 65731, Saudi Arabia; elhenawy_sci@hotmail.com; 6 Chemistry Department, Faculty of Science, Al-Azhar University, Cairo 11884, Egypt; 7 Botany & Microbiology Department, Faculty of Science, Cairo University, Giza 12613, Egypt; saoufeg@yahoo.com

**Keywords:** acyclic heterocyclic compound, trichloroisocyanuric acid, anticancer efficacy, antioxidant capacity, antifungal activity, molecular docking study

## Abstract

**Background/Objectives**: This study aimed to synthesize a novel, high-molecular-weight acyclic heterocyclic compound, compound **5**, via a one-pot reaction between Trichloroisocyanuric acid (TCCA) and ethanolamine, and evaluate its anticancer, antioxidant, and antifungal activities. **Methods:** Its complex tetrameric structure, assembled through N-N linkages, was unequivocally confirmed by a full suite of spectroscopic techniques including IR, ^1^H & ^13^C NMR, ^2^D-NMR, and high-resolution mass spectrometry (LC/Q-TOF/MS). The MTT assay was used to assess the anticancer activity of compound **5** against four different human cancer cell lines. **Results:** The findings indicate that human colon (HT29) and ovarian (OVCAR3) cancer cells were sensitive to the treatment, whereas brain (glioblastoma) (T98G) cancer cells were resistant. The most pronounced cytotoxic effect was observed in pancreatic (MiaPaCa2) cancer cells. Notably, compound **5** exhibited potent antifungal properties, achieving 100% inhibition of the pathogenic water mould *Saprolegnia parasitica* zoospores at 100 µM after 10 min. Molecular docking studies corroborated the biological data, revealing a high binding affinity for key cancer and fungal targets (Thymidylate Synthase and CYP51), providing a strong mechanistic basis for its observed activities. **Conclusions:** These findings establish compound **5** as a promising dual-action agent with significant potential as both a targeted anticancer lead and an eco-friendly antifungal for applications in aquaculture.

## 1. Introduction

Cancer is defined as uncontrolled, random tumour cell proliferation resulting from a defect in some proteins of the cell cycle [1]. This random proliferation occurs in multiple cell types, paving the way for many malignant tumors. In addition to being a serious health problem, cancer is a social and economic problem that exhausts health sectors worldwide. Approximately with 20 million new cancer cases recorded in 2022, cancer has become a major cause of increased mortality worldwide, accounting for at least one in six deaths. The mortality rate due to colorectum, pancreas, and brain cancers accounted for 9.3%, 4.8%, and 2.6%, respectively, of global cancer deaths in 2022 [2]. Despite the tremendous progress achieved in the treatment of many incurable diseases, the search for novel drugs that suppress cancer of all types remains one of the most important challenges facing modern medicine.

Free radicals are normally formed during metabolic reactions in our cells. Within the low to moderate levels, free radicals perform many important cellular functions. When their levels exceed normal limits, the cellular antioxidant system removes them and restores intracellular balance, which is called cellular homeostasis. The increase in free radicals and a weakening of the cellular antioxidant system leads to the development of oxidative stress, a key role in the pathophysiology behind many diseases such as inflammation, cancers, cardiovascular diseases, neurodegenerative disorders, allergic diseases, and diabetes [3]. Therefore, synthesis of new pharmaceutical compounds with antioxidant properties capable of inhibiting oxidative stress has become an important strategy in the treatment of many diseases.

Water moulds, e.g., Saprolegnia, cause mass mortality immediately after eggs are exposed to water during the hatching period by directly withdrawing oxygen from the egg surroundings. These water moulds first adhere to the fish eggs, which is followed by dynamic penetration into the egg. Once established, these fungi can rapidly spread to healthy eggs [4]. For decades, industry has relied on harsh chemical treatments like formalin, effective yet fraught with environmental and safety concerns. The search for a safer, “greener” alternative is not just an academic pursuit; it is a critical economic and ecological need.

Trichloroisocyanuric acid (TCCA) has antibacterial activity [5], and so it is widely used as a sterilizer in many aspects of life [6]. It is used as a highly efficient catalyst for many reactions [7]. It also participates in many chemical reactions directly and is utilized as environmentally friendly agents in mild conditions, giving products in good yields [8,9,10,11]. Studies on the anticancer effects of TCCA have not been reported in literature, but it has been used as a catalyst for synthesis of compounds with therapeutic effects against hematological malignancies [12]. Although TCCA has shown antioxidant effects in plant studies [13], we have not observed clear studies of this ability in other models.

Ethanolamine (EA) and its derivatives are used in chemical recycling of polyester [14,15]. It is also involved in some reactions such as polyethyleneimine derivative kinetics of modification of polysilicic acids, alkylation, and rearrangement and nucleophilic substitution reaction [16,17,18,19]. In the context of pharmacological and therapeutic effects, EA alone improved intestinal function and enhanced mucosal antioxidant capacity in Sprague–Dawley rats [20]. Docosahexaenoic acid (DHA) showed more anti-breast cancer activity compared to eicosapentaenoic acid (EPA) and this superiority was attributed to conversion of DHA to *N*-docosahexaenoyl ethanolamine (DHEA) [21]. A recent study has indicated that novel aza-acyclic nucleosides, synthesized starting from TCCA, exhibited antimicrobial, anticancer, and antioxidant activities [22]. Based on these results, we decided to conduct a reaction between TCCA and EA, with the hope of obtaining a new acyclonucleoside. Our compound differs from other acyclovir analogues in the nitrogen base (TCCA), which was used for the first time in our previous article [22], as well as in the ethanolamine chain. Moreover, the anticancer capacity of this novel compound was evaluated using 4 cell lines representing human colon, pancreas, ovarian, and brain cancers. The antioxidant capacity also was checked using DPPH and CUPRAC tests. Additionally, the compound’s antifungal potential was assessed, and molecular docking studies were performed, revealing strong binding interactions with key cancer-related and fungal enzymes, thereby supporting the in vitro findings and highlighting its multi-target therapeutic promise.

## 2. Results

### 2.1. Chemistry

**Scheme 1:**
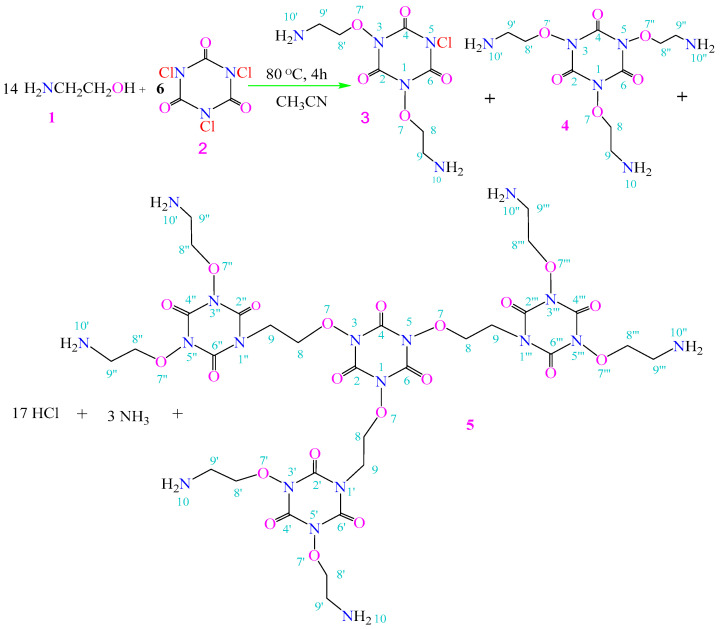
Synthesis of acyclic nucleoside derivatives **3**, **4** and **5**.

The synthesis of the novel acyclic heterocyclic compound **5** is a remarkable example of a one-pot reaction that assembles a complex, large molecule from simple, readily available starting materials. Ethanolamine reacts with trichloroisocyanuric acid, the reaction is conducted in acetonitrile at 80 °C for 4 h. Notably, it proceeds without the need for a specialized catalyst. This reaction is not perfectly selective. It yields the main target, compound **5**, in a high yield (84.19%), along with a minor mixture (11.32%) of in-termediates, Compound **3** (Bis(^1^N,^3^N)(Oxyethylamine)-^5^N-chloroisocyanuric acid) and Compound **4** (Tri(^1^N,^3^N,^5^N)(Oxyethylamine)isocyanuric acid), Scheme 1.

The inseparable mixture of compounds **3** & **4** was characterized, and their physical data are summarized in Table 1. The CI/Ms spectrum of the mixture was recorded (Figure S1). The peak for compound **3** appeared in the gas chromatogram at a retention time of 19.89 min, and the key fragments of its mass spectrum are detailed in Table 2. The peak for compound **4** appeared at 11.13 min, with its characteristic fragments listed in Table 3.

The liquid chromatography mass spectrum (LC/Q-TOF/MS) for compound **5** was also recorded (Figure S5). The Q-TOF/MS MS (ESI) showed the [M + H]^+^ ion calculated for C_30_H_49_N_18_O_21_ at 997.654075. The [M + 2H]^2+^ ion was observed at *m/z* 998.69. The key fragments of the high-resolution mass spectrum for this compound are shown in Table 4.

The infrared spectrum of compound **5** was recorded and showed the following important absorptions (KBr)cm^−1^: 3183–2960, 2935–2910, 1612, 1485, 1433, 1410, 1360, 1252, 1077, 1012. Nuclear magnetic resonance (NMR 400 MHz) spectroscopy: (^1^HNMR, ^13^C NMR, DEPT-135, ^1^H-^1^H COSY and HSQC) of compound **5** was recorded in DMSO-d6 (Figures S2–S4). ^1^HNMR (DMSO-d6) δ_H_ ppm: 2.50 (DMSO-d6, *br s*, 2.75 (18H, *t*, *J* = 4.45 Hz, -CH_2_-), 3.50 (18H, *t*, *J* = 5.42 Hz, -CH_2_-), 6.50 (12H, *br s*, -NH-C=, +H_2_O), ^13^CNMR (DMSO-d6) δ_C_ ppm: 42.64, 60.07, 154.87, DEPT δ ppm: 42.64 (-CH_2_-), 60.07 (-CH_2_-). COSY δ ppm: (^1^H/^1^H): (2.75/3.50, 6.50), (3.50/2.75), (6.50/3.50). HSQC (DMSO-d6), *δ* (ppm): (^1^H/^13^C): (2.75/42.64), (3.50/60.07).

### 2.2. Anticancer Activity

**Figure 1:**
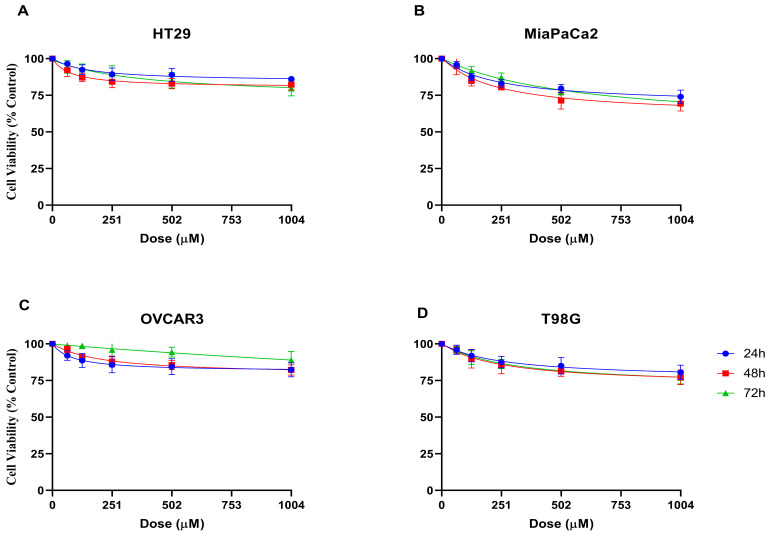
Effects of compound **5** on cell viability of HT29, MiaPaCa2, OVCAR3, and T98G cell lines. A clear, multi-panel graph (**A**–**D**) for each cell line. Each panel has distinct symbols/lines for 24 h, 48 h, and 72 h, with error bars (representing SD) clearly visible. Axes are labeled clearly: The dose of compound **5** (μM) and cell viability (%). Cytotoxic activity of compound **5** against human cancer cell lines is selective and time-dependent. Cell viability was assessed using the MTT assay after 24, 48, and 72 h of treatment with compound **5** (62.75–1004 μM). Data are presented for (**A**) HT29 (Human colon cancer cells), (**B**) MiaPaCa-2 (Human pancreatic cancer cells), (**C**) OVCAR-3 (Human ovarian cancer cells), and (**D**) T98G (Glioblastoma cancer cells) lines. Points represent the mean ± SD. Compound **5** exhibited potent, dose-dependent cytotoxicity in MiaPaCa-2 cells, with efficacy increasing over time. In contrast, T98G cancer cells showed significant resistance, with minimal reductions in viability. HT29 and OVCAR3 cancer cells were sensitive to the treatment.

In this study, the cytotoxic effects of compound **5** on different cancer cell lines were evaluated using the MTT assay at 24, 48, and 72 h of incubation. According to the results obtained, a dose-dependent decrease in cell viability was observed in all tested cell lines within the applied concentration range (Figure 1). HT29 colorectal cancer cells (Figure 1A) exhibited a moderate sensitivity to the applied doses. Cell viability showed a partial decrease, particularly at higher concentrations. MiaPaCa2 pancreatic cancer cells (Figure 1B) exhibited the most pronounced sensitivity to compound **5**. A statistically significant decrease in cell viability was observed with increasing doses, and this effect was more evident at 48 and 72 h of incubation. OVCAR3 ovarian cancer cells (Figure 1C) were also found to be sensitive to the treatment, showing a considerable reduction in viability, particularly at higher concentrations. In contrast, T98G glioblastoma cells (Figure 1D) were found to be more resistant compared to the other cell lines. Their viability showed less variation across the tested dose range, and the cytotoxic effect of compound **5** remained limited. Error bars represent the standard deviation of three independent experiments performed in triplicate.

In the HT29 cell line, a dose-dependent cytotoxic effect was observed. At the highest concentration of 1004 μM, cell viability decreased to 86.1%, 82.3%, and 79.7% at 24, 48, and 72 h, respectively. The IC_50_ values for HT29 were 149.89, 74.82, and 393.9 μM at 24, 48, and 72 h, respectively, with R^2^ values of 0.7280, 0.7972, and 0.7077.

In the MiaPaCa-2 cell line, at 1004 μM, cell viability remained at 74.1%, 69.3%, and 71.0% at 24, 48, and 72 h, respectively. The IC_50_ values were found to be 260.2 µM (R^2^ = 0.7505), 247.7 µM (R^2^ = 0.7803), and 304.3 µM (R^2^ = 0.8787), indicating a concentration-dependent cytotoxicity effect on cell viability. These results indicate that, in the MiaPaCa2 cell line, the compound exhibited a higher cytotoxic effect. In the OVCAR3 cell line, at a concentration of 1004 µM, cell viability was 82.3%, 82.2%, and 88.9% at 24, 48, and 72 h, respectively. The IC_50_ values were calculated as 84.5 µM (R^2^ = 0.6835), 204.7 µM (R^2^ = 0.8467), and 4535 µM (R^2^ = 0.5833). In the T98G cell line, a notable dose-dependent de-crease in viability was observed. At 1004 μM, cell viability was 80.5%, 77.2%, and 76.9% at 24, 48, and 72 h, respectively. The IC_50_ values for T98G were 261.2, 248.7, and 305.5 μM for the corresponding time points, with R^2^ values of 0.7505, 0.7803, and 0.8787, indicating a good correlation between dose and response (Table S1).

### 2.3. Antioxidant Activity

The antioxidant activity of compound **5** was evaluated using the DPPH and CU-PRAC assays. In the DPPH assay (Table S2), compound **5** demonstrated dose-dependent radical scavenging activity. At 1004 µM, the inhibition rate of compound **5** was 22.02 ± 0.93%, significantly lower than the positive control, ascorbic acid (AscA), which exhibited an inhibition rate of 67.13 ± 4.51% at the same concentration. As the concentration of compound **5** decreased, the inhibition rate also decreased, with 62.75 µM resulting in only 6.63 ± 2.97% inhibition. The calculated IC_50_ value for compound **5** was 115.6 µM (R^2^ = 0.8195), while for AscA, the IC_50_ value was 38.5 µM (R^2^ = 0.9067), indicating that compound **5** had a weaker radical scavenging effect compared to AscA.

In the CUPRAC assay (Table S2), compound **5** also exhibited lower reducing capacity compared to AscA. At 1004 µM, the absorbance of compound **5** was 0.135 ± 0.005, whereas AscA showed an absorbance of 1.768 ± 0.176 at the same concentration. At lower concen-trations (62.75 µM), compound **5** showed an absorbance of 0.164 ± 0.01, indicating a min-imal cupric ion-reducing capacity. The IC_50_ value for compound **5** in the CUPRAC assay was estimated to be approximately 5.08 × 10^16^ µM (R^2^ = 0.1330), indicating extremely weak reducing activity, whereas AscA had a much lower IC_50_ of 1527 µM (R^2^ = 0.9713). These results suggest that compound **5** exhibits limited antioxidant potential in comparison to the positive control AscA.

**Figure 2:**
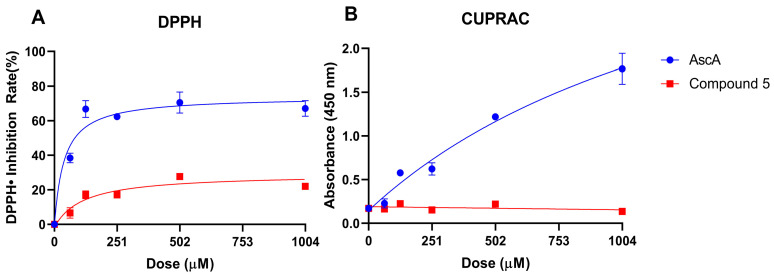
Antioxidant activity of Compound **5** compared to AscA in DPPH and CUPRAC assays. A clear bar graph with two sets of bars (Compound **5** and Ascorbic Acid), for each concentration in the DPPH and CUPRAC assays. Error bars are included. The y-axes are labeled appropriately: DPPH Scavenging Activity (%) and CUPRAC Absorbance (450 nm). Compound **5** exhibits moderate antioxidant capacity in vitro. (**A**) DPPH radical scavenging activity and (**B**) Cupric ion reducing antioxidant capacity (CUPRAC) of compound **5** compared to ascorbic acid (AscA, positive control). Samples were tested at concentrations ranging from 62.75 to 1004 µM. Values represent the mean ± SD. Compound **5** demonstrated moderate, dose-dependent activity in the DPPH assay but was significantly less effective than AscA in both assays, indicating limited direct antioxidant potential.

In general, AscA (positive control) shows a significantly strong antioxidant effect in both assays, while compound **5** shows a lower activity, suggesting that compound **5** has a more limited antioxidant potential (Figure 2). Error bars represent the standard deviation of three independent experiments performed in triplicate.

### 2.4. Antifungal Activity

Table 5 presents compelling evidence that compound **5**, a novel TCCA derivative, could be a key player in this search.

Table 5 presents compelling evidence that compound **5**, a novel TCCA derivative, could be a key player in this search. The antifungal assay was performed in triplicate, and results are expressed as mean ± SD. At the lowest concentration (20 µM) and shortest exposure (3 min), compound **5** inhibited only 8.42 ± 2.11% of *Saprolegnia parasitica* zoospores. Increasing exposure time to 5 min at the same concentration improved inhibition to 12.42 ± 3.51%, and at 10 min, inhibition reached 18.42 ± 2.11%. With higher concentrations, both the speed and extent of inhibition increased markedly. At 60 µM, inhibition rose to 27.02 ± 4.51% (3 min) and 65.22 ± 5.61% (10 min). The most dramatic results were observed at 100 µM, where inhibition reached 53.40 ± 5.22% after 3 min, 88.50 ± 7.30% after 5 min, and complete inhibition (100 ± 0.0%) after 10 min.

These results show a clear, reproducible dose- and time-dependent relationship, demonstrating that compound **5** achieves total zoospore inhibition at 100 µM within 10 min, significantly surpassing the positive control formalin, which produced only 14.2 ± 2.6% inhibition at the same time point.

### 2.5. Molecular Docking Studies

To elucidate the potential mechanisms behind the observed biological activities, molecular docking studies were performed against two key targets: human Thymidylate Synthase (TS, PDB: 1HZW), a critical enzyme in cancer cell proliferation, and fungal Lanosterol 14α-demethylase (CYP51, PDB: 5V5Z), a cornerstone of ergosterol biosynthesis.

#### 2.5.1. Molecular Docking of Compound 5 into the Active Site of Thymidylate Synthase

The docking simulations revealed that compound **5** successfully docked into the active site of TS. The calculated binding energy for compound **5** was −5.45 kcal/mol. For comparison, the co-crystallized reference inhibitor, a raltitrexed analogue (Gol.), exhibited a binding energy of −3.41 kcal/mol under the same conditions (Table 6).

To elucidate the molecular mechanism underlying the observed cytotoxic activity of compound **5**, we performed molecular docking studies against human Thymidylate Synthase (TS, PDB ID: 1HZW [23]). TS is a critical enzyme in the de novo pyrimidine nucleotide biosynthesis pathway, catalyzing the reductive methylation of deoxyuridine monophosphate (dUMP) to deoxythymidine monophosphate (dTMP) [24]. As dTMP is an essential precursor for DNA synthesis, the inhibition of TS leads to a “thymineless death,” effectively halting cell proliferation, particularly in rapidly dividing cancer cells. This makes TS a well-validated and clinically relevant target for anticancer drug development, with established inhibitors like 5-fluorouracil and raltitrexed [25]. The study aimed to predict the binding affinity of compound **5**, analyze its binding mode within the TS active site, and compare its interactions with those of a known reference inhibitor to rationalize its potent biological activity. The docking simulations, performed using AutoDock Vina, provided binding energy scores that quantify the binding affinity between the ligands and the TS active site. The results for compound **5** and the reference inhibitor are summarized in Table 6.

The computational results predict that compound **5** exhibits a significantly stronger binding affinity (−5.454 kcal/mol) for the TS active site compared to the known inhibitors (−4.153 kcal/mol, and −3.984 kcal/mol), respectively.

**Figure 3:**
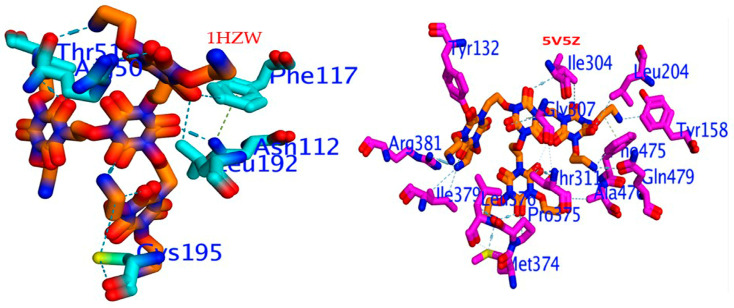
High-affinity binding of compound **5** (orange carbons) to therapeutic targets, including active site of hTS (PDB: 1HZW) and CYP51 (PDB ID: 5V5Z). Key interacting residues (magenta carbons) are shown as sticks. Hydrogen bonds and water-mediated interactions are represented by light-blue dashed lines.

The binding orientation is anchored by a combination of hydrogen bonds, hydrophobic interactions, and water-mediated contacts. Molecules from H-bond networks, including donors and acceptors, are fully exploited. A critical H-bond is formed between the carbonyl oxygen of one of the 1,3,5-triazine-2,4,6-trione rings and the backbone amide of ARG50. Furthermore, the terminal primary amine groups on the oxyethylamine side chains engage in strong hydrogen bonding with the side-chain carboxylate of ASN112, which can also act as an ionic interaction (salt bridge) if the amine is protonated at physiological pH. The binding is substantially stabilized by hydrophobic interactions. The large, planar indole ring of PHE117 engages in a favorable π-π stacking interaction with one of the triazine rings of Compound **5**. This interaction is crucial for correctly positioning the ligand deep within the pocket. The aliphatic side chain of LEU192 (Figure 3). Notably, the binding of compound **5** is further stabilized by several bridging water molecules. These water-mediated H-bonds create a highly stable network, linking the carbonyl oxygens of the ligand to the side-chain amine of CYS195 and other polar residues in the active site. This ability to favorably interact with and organize structural water molecules is a hallmark of high-affinity inhibitors [26].

#### 2.5.2. Molecular Docking Analysis into the CYP51 Active Site

Molecular docking simulations were conducted to predict the binding affinity and interaction profile of compound **5** within the CYP51 active site, with a known azole-class reference inhibitor 2-[(2R)-butan-2-yl]-4-{4-[4-(4-{[(2R,4S)-2-(2,4-dichlorophenyl)-2-(1H-1,2,4-triazol -1-ylmethyl)-1,3-dioxolan-4-yl]methoxy}phenyl)piperazin-1-yl]phenyl}-2,4-dihydro-3H-1,2,4-triazol-3-one (1YN) included for comparison. Compound **5** also demonstrated a high binding affinity for the fungal CYP51 active site, with a calculated binding energy of −9.87 kcal/mol. This was superior to the binding energy of the co-crystallized azole reference inhibitor (1YN), which was −9.55 kcal/mol (Table 6). Analysis of the lowest-energy pose indicated critical interaction, with one of the triazine nitrogen atoms of compound **5** positioned at a distance of approximately 2.2 Å from the heme iron, suggesting direct coordination similar to established azole antifungals.

### 2.6. Quantum Chemical Analysis: Electronic Structure and Reactivity

**Figure 4:**
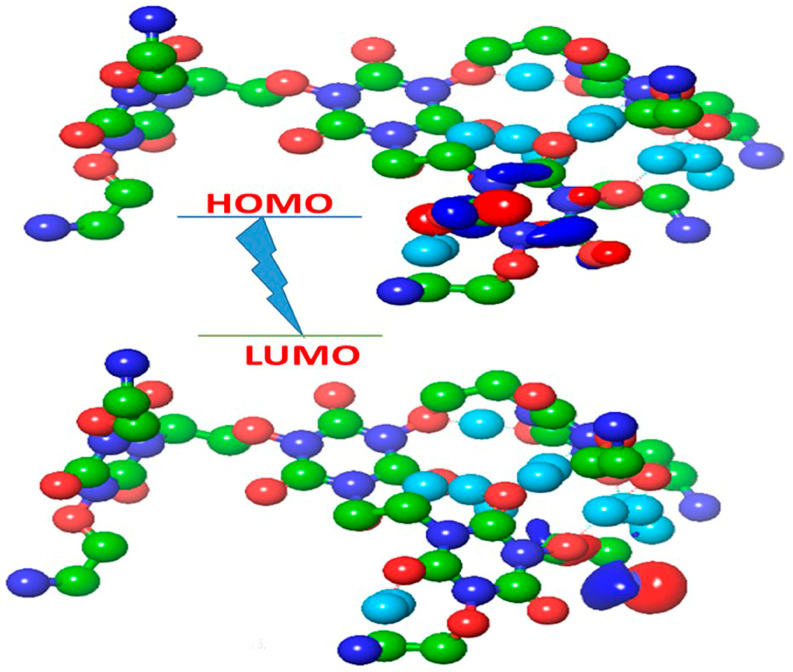
FMO (HOMO and LUMO) surfaces of compound **5** using DFT at B3LYP/6-311G++(d,p) level. A consistent and intuitive color scheme is used (e.g., red for negative phase, green for positive phase).

To gain a fundamental understanding of the structure-activity relationship (SAR) at the electronic level, we employed Density Functional Theory (DFT) calculations. The electronic properties and global reactivity indices of the target compound **5** and its precursors (**3** and **4**) were computed at the B3LYP/6-311G++(d,p) level of theory. This analysis provides a quantitative framework to explain why compound **5** exhibits superior biological efficacy compared to its simpler analogues. The shapes and energies of the Frontier Molecular Orbitals the Highest Occupied Molecular Orbital (HOMO) and the Lowest Unoccupied Molecular Orbital (LUMO) are paramount in dictating a molecule’s reactivity and its ability to interact with biological receptors. The LUMO, which indicates the most probable site for accepting an electron (i.e., the center of nucleophilic attack), is distinctly localized on one of the 1,3,5-triazine-2,4,6-trione rings and its adjacent oxyethylamine linker. This localization effectively designates this region as the molecule’s “electrophilic warhead.” In a biological context, this site is highly susceptible to attack from electron-rich, nucleophilic residues (e.g., Cys, Ser, His, or the carboxylate of Glu/Asp) within an enzyme’s active site, or from the N7 atom of guanine in DNA. This targeted electrophilicity is a key driver of covalent or strong non-covalent inhibition (Figure 4). Conversely, the HOMO, representing the region most capable of donating an electron, is delocalized over the more electron-rich portions of the molecule, specifically the extensive network of the other triazine rings and the numerous nitrogen and oxygen lone pairs of the appended side chains. This diffuse HOMO distribution suggests the molecule’s ability to participate in numerous charge-transfer and hydrogen.

To quantify the differences in chemical reactivity between compounds **3, 4,** and **5**, a series of global reactivity indices were calculated from their FMO energies (Table 7). These descriptors provide a powerful lens through which to interpret their biological activity.

The data in Table 7 reveal a clear and dramatic trend. Energy Gap (ΔƐ): compound **5** possesses a drastically smaller HOMO-LUMO energy gap (ΔƐ = 1.60 eV) compared to its precursors.

### 2.7. Protein Dynamics and Mechanism of Inhibition: A Normal Mode Analysis (NMA) Perspective

To move beyond the static picture provided by molecular docking, we employed Normal Mode Analysis (NMA) on the ligand-protein complexes. NMA is a powerful computational technique that probes the intrinsic, low-frequency collective motions of a protein, which are often essential for its biological function, including substrate binding, catalysis, and allosteric regulation [27]. By analyzing the dynamics of the enzyme in complex with compound **5**, we can gain crucial insights into the stability of the docked pose and the molecular mechanism of inhibition.

#### 2.7.1. NMA of the Compound **5**-1HZW Complex

**Figure 5:**
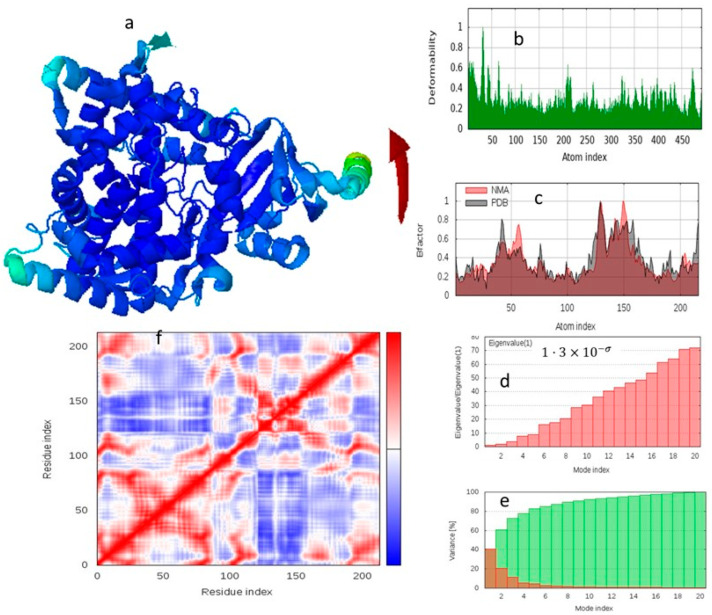
The mechanism of dynamic inhibition (NMA) of 5-1HZW complex. (**a**) Overview of compound **5**-complex. (**b**) Deformability plot along the protein residue index, showing peaks corresponding to high flexibility in the substrate access channel loops. (**c**) 3D visualization of B-factors (blue: rigid, red: flexible), highlighting the rigid active site and flexible external loops. (**d**) Covariance matrix of residue motions, illustrating large-scale correlated (red) and anti-correlated (blue) motions. (**e**) Cumulative variance plot, showing that the first 20 low-frequency normal modes account for over 90% of the system’s dynamics. (**f**) Representation of a dominant low-frequency mode, illustrating the collective “breathing” motion that is restricted upon binding of compound **5**, which acts as a “molecular plug”.

Inference on inhibition mechanism: TS catalysis is known to involve a significant conformational change where a flexible C-terminal loop closes over the active site after substrate binding to shield the reaction from solvent. The NMA identifies this loop as one of the most dynamic regions. Compound **5** acts as a “molecular brace” or “wedge.” By binding tightly within the active site and forming strong interactions with key residues like PHE117 and ASN112 (as per docking), it prevents the necessary closure of this C-terminal loop. It effectively locks the enzyme in an open, catalytically incompetent conformation. The mechanism is therefore not merely a competitive inhibition but a dynamic arrest of the enzyme’s functional cycle (Figure 5).

#### 2.7.2. NMA of the Compound **5**-CYP51 (5V5Z) Complex

**Figure 6:**
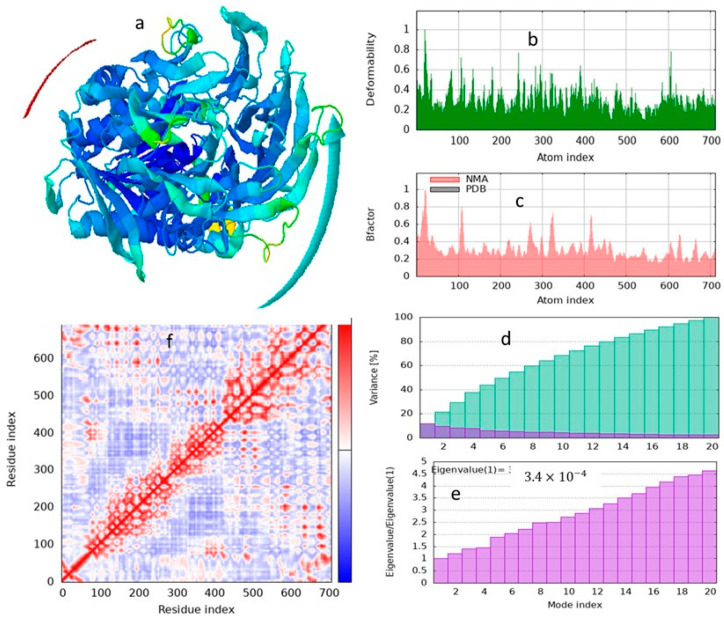
The mechanism of dynamic inhibition (NMA) of 5-5V5Z complex. (**a**) Overview of compound **5**-complex. (**b**) Deformability plot along the protein residue index, showing peaks corresponding to high flexibility in the substrate access channel loops. (**c**) 3D visualization of B-factors (blue: rigid, red: flexible), highlighting the rigid active site and flexible external loops. (**d**) Covariance matrix of residue motions, illustrating large-scale correlated (red) and anti-correlated (blue) motions. (**e**) Cumulative variance plot, showing that the first 20 low-frequency normal modes account for over 90% of the system’s dynamics. (**f**) Representation of a dominant low-frequency mode, illustrating the collective “breathing” motion that is restricted upon binding of compound **5**, which acts as a “molecular plug”.

The NMA results for the CYP51-compound **5** complex reveal a highly dynamic system with distinct regions of rigidity and flexibility (Figure 6a). Deformability and B-factor Analysis, the deformability plot (Figure 6b) highlights several peaks corresponding to regions of high atomic mobility. These peaks are primarily located in the loop regions that form the entrance to the substrate access channel leading to the heme-containing active site. The 3D B-factor visualization (Figure 6c) corroborates this, showing these external loops colored in yellow/green (flexible), while the core of the enzyme, particularly the residues forming the active site pocket, are colored blue (rigid). The reasonable correlation between the NMA-calculated B-factors (red line) and the experimental PDB B-factors (black line) validates the computational model, confirming that it accurately captures the protein’s intrinsic dynamics (Figure 6).

### 2.8. In Silico ADMET and Drug-likeness Prediction

**Figure 7:**
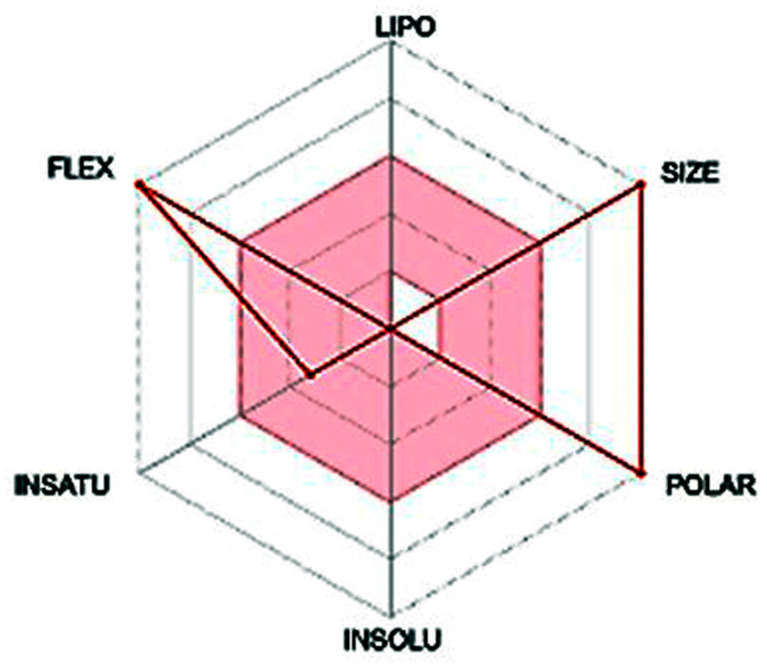
Drug-likeness evaluation of compound **5** using a bioavailability radar. The plot compares six key physicochemical properties of the compound (pink area) against the ideal space for oral bioavailability (light pink). The compound’s profile falls outside the desirable range for lipophilicity (LIPO), size (SIZE), polarity (POLAR), insolubility (INSOLU), insaturation (INSATU), and flexibility (FLEX), consistent with its large, polar macrocyclic structure.

To preliminarily assess the pharmacokinetic and toxicity profile of the lead compound **5**, a computational analysis was performed using the Swiss ADME web tool. The key parameters are summarized in Table 8, and the physicochemical space is visualized in the bioavailability radar chart (Figure 7).

## 3. Discussion

### 3.1. Chemistry

The inseparable twin compounds **3** & **4,** shown in Table 1, are the first clue. This information tells us that these are not just trace impurities; they are a significant side-products of the reaction, a supporting cast that is crucial to understanding the full story. The physical evidence (Rf and M.P.) is as follows. Both compounds share an identical Rf value of 0.28 on the TLC plate. The Rf value is a measure of polarity; identical values mean these two molecules 3, and 4 behave almost identically, like twins moving in perfect unison. This similar polarity makes separating them by standard chromatography a near-impossible task. Their shared decomposition range (252–258 °C) further cements their close relationship. While the compounds **3** and 4 act like twins, their molecular formulas reveal their true, distinct identities. Our di-substituted intermediate 3 still carries a reactive chlorine atom, while compound **4** has the fully substituted, non-chlorinated intermediate.

**Scheme 2:**
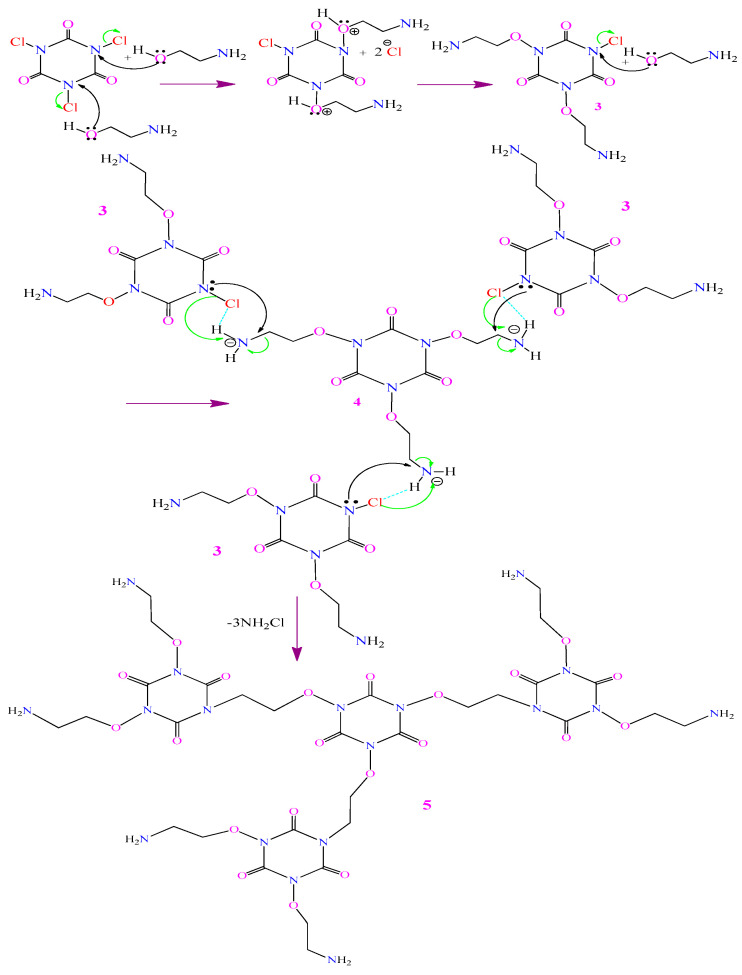
Formation of compound **5**.

The story is clear: these are two closely related siblings born from the same reaction pathway, differing only by a single substitution. This subtle difference is not enough to alter their overall polarity, making them “chromatographic twins” that co-exist in the 11.32% mixture. Turn our attention to the main compound, the molecule our synthesis was designed to create. Unlike the twins, compound **5** stands alone with a distinct Rf value of 0.2. This lower value indicates it is significantly more polar than the intermediates. This makes perfect sense: it is a much larger molecule, packed with a higher density of polar amine, carbonyl, and ether groups, causing it to “stick” more tightly to the TLC plate. Its sharp decomposition point at 245 °C confirms it is a distinct and pure substance. The dramatic leap from the ~300 g/mol intermediates to this massive structure is the most powerful piece of evidence in Table 1. It tells us that compound **5** is not the result of a simple substitution, but a magnificent assembly. The numbers quantitatively prove that the smaller intermediates have coupled together, just as the mechanism in Scheme 2 proposes, to form a single, giant, and well-defined macromolecule. In conclusion, Table 1 is far more than a dry collection of data. It is the quantitative proof of our synthetic narrative. It introduces us to the inseparable intermediates, highlights the stunning efficiency of the main reaction, and, most importantly, provides the numerical fingerprint of a molecular giant, validating the entire proposed mechanism of its assembly.

The formation of compound **5** is a multi-step process involving nucleophilic substitution and a subsequent coupling reaction. The detailed mechanism is proposed in Scheme 2. The mechanism in Scheme 2 rightly begins with the initial reaction between trichloroisocyanuric acid TCCA and ethanolamine. The primary -NH_2_ of ethanolamine is a potent nucleophile. It attacks the electron-deficient nitrogen atoms of the TCCA ring. These nitrogens are electrophilic because they are bonded to a highly electronegative chlorine atom, which withdraws electron density. The classic nucleophilic substitution at a N center. The amine’s lone pair forms a new N-N bond (initially, then rearranges), and the N-Cl bond breaks, expelling a (Cl^−^) as an excellent leaving group. This substitution can occur one, two, or three times on the TCCA ring, leading to a mixture of products. Scheme 2 explicitly shows the formation of the fully substituted compound **4** (where all three chlorines are replaced) and the di-substituted compound **3** (which retains one reactive N-Cl bond). The presence of both intermediates in the reaction pot is essential for the next phase. Phase II includes critical N-N coupling and assembly. The nucleophile acts as the terminal -NH_2_ group on one of the oxyethylamine side chains of compound **4**.

The target of the attack is the electron-deficient nitrogen atom of the N-Cl bond on compound **3**. This is the only remaining electrophilic site of its kind. The lone pair of electrons on the amine nitrogen of compound **4** attacks the N-Cl nitrogen of compound **3**. This attack forms the crucial N-N bond that links the two triazine ring systems together. Simultaneously, the N-Cl bond cleaves, with the chlorine leaving as Cl^−^. The result is a cationic intermediate where the attacking nitrogen now bears a positive charge. However, the central compound **4** molecule has three identical nucleophilic side chains. Therefore, a single molecule of compound **4** can sequentially react with three separate molecules of compound **3**. This threefold reaction perfectly explains the 1:3 stoichiometry proposed in Scheme 2 and leads directly to the final, large, and highly symmetrical architecture of compound **5**.

In conclusion, Scheme 2 provides a compelling and chemically sound narrative for the formation of compound **5**. The proposed pathway highlights a sophisticated one-pot synthesis where the initial products (intermediates **3** and **4**) become the reactants for a final, convergent assembly step. The cornerstone of this entire process is the dual reactivity of the starting materials, leading to the formation of a nucleophilic core (compound **4**) and an electrophilic linker (compound **3**), which then combine through a critical N-N bond-forming reaction to build the final macromolecular structure.

#### 3.1.1. Confirmation of the Structure of Compound **5**

##### The Fingerprint of Symmetry: Deconstructing the Structure of Compound **4**

**Scheme 3:**
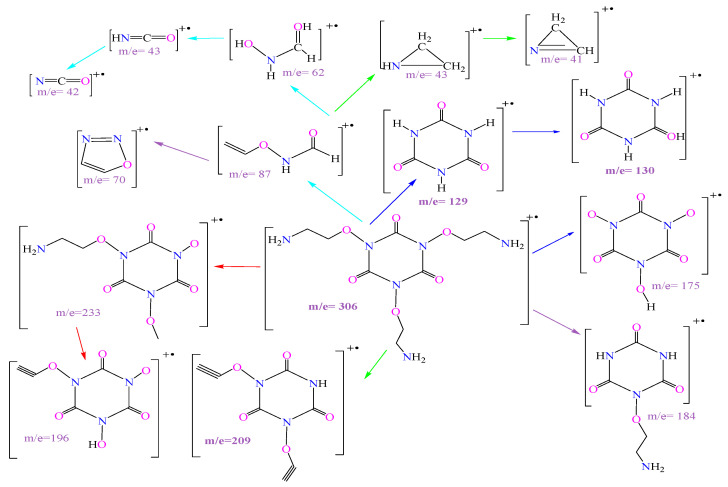
The fragmentation pattern of compound **4**.

If compound **3** was a case of asymmetric evidence, compound **4,** the fully substituted, non-chlorinated intermediate, presents us with a beautiful case of molecular symmetry. Its fragmentation pattern, shown in Table 3 and Scheme 3, is not random; it is a direct reflection of its perfectly balanced architecture. Our molecule of interest is Tri(^1^N,^3^N,^5^N)(Oxyethylamine)isocyanuric acid, with a mass of 306 g/mol. The text notes its instability, but the appearance of a related ion at *m*/*z* 308 (likely [M + 2H]^+^) confirms we are in the correct mass range. Critically, it’s even mass (306) fits perfectly with the nitrogen rule for a molecule with an even number of nitrogen atoms (six), providing a beautiful contrast to its chlorinated sibling, compound **3**. The base peak for compound **4** is at *m*/*z* 129. This fragment is the bare isocyanuric acid ring, having shed all three of its identical side chains.

The fact that this is the most stable and abundant fragment is a powerful testament to the molecule’s design. In a highly symmetrical molecule, the three identical side chains are energetically equivalent. It is therefore highly probable that the molecule will fragment by shedding all three of these “limbs” in a symmetrical collapse, leaving behind the stable central core. The mass of 129 perfectly matches this C_3_H_3_N_3_O_3_ core. This peak is the definitive fingerprint of the central triazine scaffold. The rest of the pattern reads like a story of identical pieces breaking away. The loss of one unit (*m*/*z* 233), this peak is crucial. The mass difference between the parent ion (306) and this fragment (233) is 73 daltons. This corresponds precisely to the mass of one complete oxyethylamine side chain. It is the clearest possible evidence for the existence and mass of the repeating structural unit. Fragments of the Side Chain (*m*/*z* 41, CH=N-CH_2_): Unlike in Compound **3**, we now see fragments that can only come from the breakdown of the ethylamine portion of the side chain, further confirming its structure. The building block of the ring (*m*/*z* 43, HN=C=O): Once again, we see the isocyanate fragment, the fundamental building block of the ring, confirming the underlying chemistry common to both intermediates.

Finally, the fragmentation pattern in Scheme 3 is a symphony of symmetry. The symmetrical collapse to the core at *m*/*z* 129, the clean loss of a single 73-dalton side chain to give *m*/*z* 233, and the fragments from both the ring and the chains all work in concert. They paint an elegant and undeniable picture of a central isocyanuric acid core symmetrically decorated with three identical oxyethylamine side chains, providing an unequivocal structural confirmation for compound **4**.

##### Liquid Chromatography Mass Spectrum (LC/Q-TOF/MS) (Figure S5)

**Scheme 4:**
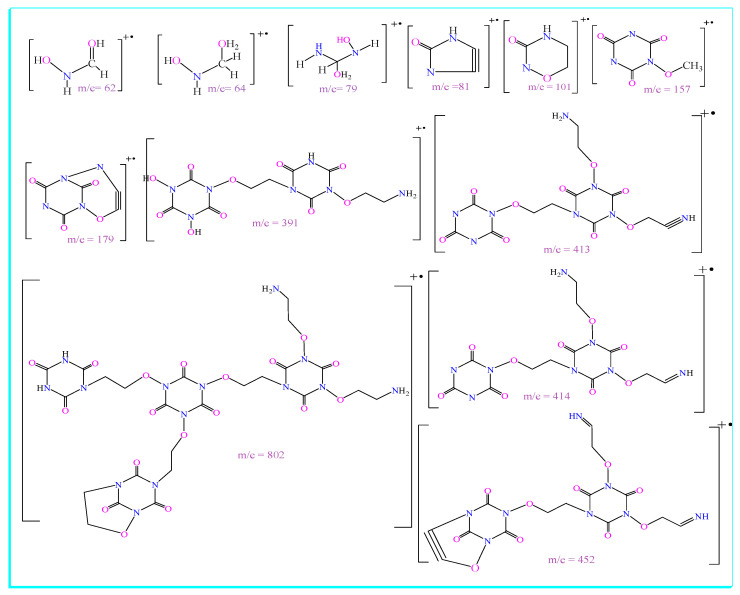
The fragmentation pattern of compound **5**.

The ultimate test of any synthesis is the characterization of its final product. For a molecule as large and intricate as compound **5**, this is no small feat. It requires a symphony of analytical techniques, each playing its part to build a case so strong it becomes undeniable. The combination of it’s mass spectrum (Table 4), the proposed mechanisms (Scheme 2), and its fragmentation pattern (Scheme 4) provides just such a case a beautiful, convergent proof of a molecular masterpiece.

The data in Table 4 is profound compound **5**, is a molecular behemoth with a calculated formula of C_30_H_48_N_18_O_21_ and an exact mass of 996.53 g/mol. The first clue is the absence of a simple protonated ion at *m*/*z* 997. For such a large molecule, rich with basic nitrogen atoms, this is not surprising. Instead of taking on a single proton, it readily accepts two, forming a doubly charged ion. This is where the key evidence lies: the peak at *m*/*z* 998.69, corresponding to the [M+2H]^2+^ ion. This doubly charged species confirms a molecular weight of approximately 997 g/mol, providing unambiguous proof that we have successfully created the massive target molecule. The Q-TOF/MS instrument gives us the precision to move beyond approximation to certainty, confirming the molecular formula and setting the stage for understanding its architecture. Scheme 4 is the high-level strategy. It masterfully simplifies the complex reaction into its core logic: a convergent assembly. It proposes that the final structure is not built by a linear, step-by-step addition, but by the joining of pre-formed, specialized building blocks. The “Core” (compound **4**), A central hub with three nucleophilic “hands” (the -NH_2_ groups). The “Linker” (compound **3**): Three identical arms with electrophilic “hooks” (the N-Cl bonds).

The most stable shard (The base peak, *m*/*z* 413): In the violent chaos of the mass spectrometer, the most stable pieces survive best. Table 4 shows the most intense peak is at *m*/*z* 413. Scheme 4 reveals what this is: A large fragment consisting of one of the “linker” arms still attached to a piece of the central core. Its large size and resonance-stabilized structure explain its high abundance, and its mass is a perfect fingerprint of the core-linker connection. The other fragments are the supporting cast of clues. *m*/*z* 802: This massive fragment represents the nearly-intact parent molecule after cleanly losing one of its three arms. The mass difference confirms the mass of a single arm unit and proves that the molecule is indeed built from these cleavable, repeating structural motifs. *m*/*z* 452: This fragment is a key marker for the central core. It represents the original “core” molecule (compound **4**) after it has undergone the N-N coupling but subsequently lost one of its side chains. The smallest clues (*m*/*z* 157, 79, etc.): These smaller fragments represent the further breakdown of the arms and side chains, confirming their own internal structures (the oxyethylamine linkage and the triazine ring).

Individually, each piece of evidence is strong. Together, they are undeniable. The mass spectrum (Table 4) provides the definitive molecular formula. The mechanisms (Scheme 4) provide a chemically sound and elegant explanation for its high-yield formation. And the fragmentation pattern (Scheme 4) serves as the final confirmation, a molecular dissection that reveals an internal architecture perfectly matching the one predicted by the mechanism. This is the hallmark of great scientific work: A self-consistent, multi-layered narrative where every piece of data supports and enriches the others, leaving no doubt as to the identity of our molecular masterpiece, compound **5**.

##### Infrared (IR) Spectroscopy

IR spectroscopy confirms the presence of the key functional groups in compound **5**. Broad bands characteristic (3183–2960 cm^−1^) of N-H stretching in the primary amine (-NH_2_) groups, indicating hydrogen bonding. A strong absorption (1612 cm^−1^) for the carbonyl (C=O) stretching within the triazine rings. Bending vibrations for the -CH_2_- groups at 1485, 1433 cm^−1^. Stretching vibrations for the C-O and C-N-O linkages (1252 cm^−1^ and 1077 cm^−1^), respectively, confirming the presence of the oxyethylamine side chains. From the IR spectrum it has been noticed the presence of broader medium intensity bands at 3183 -2960 cm^−1^ for -NH_2_ with hydrogen bonds. 2935–2910 cm^−1^ -CH SP^3^ stretching 1612 cm^−1^ for -CO- in TCCA ring, 1485, 1433 cm^−1^ for bending -CH_2_-, –NH-,1410, 1360 cm^−1^ for bending -C-C-, 1252 for bending C-O- and 1077 for bending -C-N-O-.

##### Nuclear Magnetic Resonance (NMR) Spectroscopy (Figures S2–S4)

The NMR spectra are particularly insightful due to the high symmetry of the molecule, which simplifies the signals.

^1^H NMR (Figure S2)

The δ 6.50 ppm (12H, broad singlet) signal corresponds to the twelve protons of the six-terminal primary amine (-NH_2_) groups. Its broadness is typical for amine protons, and its disappearance upon adding D_2_O confirms these are exchangeable protons. The signal at **δ** 3.50 ppm (18H, triplet) integrates to 18 protons and is assigned to the nine methylene groups bonded to oxygen (-CH_2_-O-). δ 2.75 ppm (18H, triplet), this signal also integrates to 18 protons and is assigned to the nine methylene groups bonded to the triazine ring nitrogen (-CH_2_-N<). The high integration values (12H, 18H, 18H) are crucial evidence for the highly symmetrical, tetrameric structure.

^13^C NMR and DEPT-135 (Figure S3)

δ 154.87 ppm Signal for the carbonyl carbons (C=O) in the triazine rings. In the DEPT-135 spectrum, this peak is absent, confirming it is a quaternary carbon (a carbon with no attached protons). δ 60.07 ppm: Signal for the methylene carbons attached to oxygen (-CH_2_-O-). δ 42.64 ppm: Signal for the methylene carbons attached to nitrogen (-CH_2_-N<).

^2^D NMR (COSY and HSQC—Figure S4)

HSQC experiment definitively links the proton signals to their directly attached carbons. The proton at δ 3.50 ppm correlates with the carbon at δ 60.07 ppm. The proton at δ 2.75 ppm correlates with the carbon at δ 42.64 ppm. This provides unambiguous proof of the assignments for the methylene groups. COSY (^1^H-^1^H correlation), A cross-peak is observed between the signals at δ 2.75 ppm and δ 3.50 ppm. This shows that these two types of protons are on adjacent carbons, confirming the connectivity of the -CH_2_(N)-CH_2_(O)- fragment in the oxyethylamine side chains. This result has been reported in some compounds similar to ours, as well as in some acyclovir analogues [22,28,29]. From H-H COSY spectrum it has been noticed that δ_H_: 2.75 ppm of methylene groups connected with -NH_2_ which has δ_H_: 6.50 ppm. ROSY spectrum gave indications that the hydrogens of the methylene atoms were bonded to each other only.

In conclusion, the combination of mass spectrometry confirming the molecular formula, IR identifying the functional groups, and a full suite of NMR techniques establishing the precise atom-to-atom connectivity and high degree of molecular symmetry, provides overwhelming and conclusive evidence for the proposed structure of compound **5**.

### 3.2. Anticancer Activity

In this study we synthesized acyclo nucleosid (acyclovir analogue) which consists of a heterocycle (nitrogen base) and an open chain containing a nitrogen atom. Although some studies have reported anticancer effects of compounds close in chemical structure to compound **5** [30,31], we have not observed studies evaluating the anticancer effects of compounds synthesized starting from TCCA and ethanolamine. Benedetti et al. [30] attributed the anticancer effects of acyclovir against NCI-H1975 lung cancer cells to acyclovir’s role in mitochondrial DNA damage and activation of caspase-3, which triggers apoptosis in cancer cells. Chemically, we think that the presence of nitrogen atoms in the TCCA ring, as well as to the amine group in the open chain, contributed to demonstrating the anticancer activity of compound **5**. In this context, ethanolamine enhances DHEA ability as anti-breast cancer agent [21].

In Figure 1A,B, we see the classic downward trend of a dose-response curve. As the concentration of compound **5** increases, cell viability steadily decreases. This is the fundamental hallmark of a bioactive compound. In the HT29 cell line, cell viability was only marginally affected by increasing doses, with approximately 79% viability observed at 72 h; whereas in MiaPaCa2 cells, viability decreased to about 71% at 72 h. This is reinforced by the IC_50_ values, which are lowest at the 48-h mark for both cell lines (74.82 µM for HT29 and 248.7 µM for MiaPaCa2), indicating cytotoxicity efficacy after two days of exposure. This suggests that the molecular target of compound **5**, as explored in the docking studies (e.g., Thymidylate Synthase), might be more critical or more accessible in colon and pancreatic cells. This selective activity is the holy grail of drug development, hinting at a potential therapeutic window. The MiaPaCa-2 (pancreatic cancer) cell line exhibited the highest sensitivity to the treatment. Cell viability decreased to approximately 71% at the highest concentration, with an IC_50_ value of 304.3 μM at 72 h. In contrast, OVCAR-3 (ovarian cancer) cells showed moderate response, as their viability remained within the range of 82.3–88.9% even at higher concentrations.

In stark contrast, the brain (glioblastoma) (T98G) cancer cells tell a story of resistance suggesting that these cell lines may possess robust mechanisms to either metabolize, expel, or bypass the effects of compound **5**.

Taken together, these dose-response curves paint a complete picture. Compound **5** is not a “magic bullet” that kills all cancer cells indiscriminately. Instead, it is a promising lead compound with clear, selective activity against pancreatic cell lines. Its time-dependent efficacy and preferential targeting suggest a specific mechanism of action that is highly relevant in this cancer but less so in brain (glioblastoma) cancer. This selective profile is far more valuable than broad toxicity, providing a clear path forward for future research to understand the molecular basis of this sensitivity and optimize compound **5** as a targeted agent for the cancers it affects most.

### 3.3. Antioxidant Activity

Although some studies have reported antioxidant effects of TCCA in plants [13] and ethanolamine in rats [20], there are no studies evaluating the antioxidant capacity of the compounds synthesized starting from TCCA and ethanolamine. A previous study demonstrated that some acyclovir analogues have good to moderate antioxidant activity in vitro studies [32]. Undoubtedly, in vivo studies provide more comprehensive visualization of antioxidant capacity for synthesized compounds or natural products compared to in vitro studies. Although osajin showed a weak antioxidant effect in vitro study [33], it exhibited a potent antioxidant effect in vivo investigations [34,35]. These paradoxes prompt us to conduct a broader investigation into the antioxidant properties of compound **5** using in vivo studies.

Compound **5** is capable of scavenging a pre-formed radical (DPPH) to a moderate degree, but it almost completely lacks the ability to act as a reducing agent (CUPRAC). This suggests a specific structural feature may be responsible for its modest DPPH activity perhaps a sterically accessible hydrogen atom that can be donated while it lacks the easily oxidizable electronic structure (like the enediol system in ascorbic acid) required for potent reduction. Ultimately, these findings suggest that the primary biological effects of compound **5**, such as its promising anticancer activity, are not driven by direct antioxidant action. It is not a general-purpose molecule for combating oxidative stress. Instead, its efficacy almost certainly stems from a more specific mechanism, such as the inhibition of key enzymes like Thymidylate Synthase, as explored in the docking studies. This work successfully decouples the compound’s antioxidant potential from its cytotoxic potential, providing a crucial focus for future research.

### 3.4. Antifungal Activity

In the world of aquaculture, *Saprolegnia parasitica* is a relentless pathogen enveloping fish eggs, causing devastating losses in hatcheries worldwide [4]. For decades, industry has relied on harsh chemical treatments like formalin. The search for a safer, “greener” alternative is not just an academic pursuit; it is a critical economic and ecological need.

The parent molecule, TCCA, is already widely used as a water disinfectant precisely because it is effective at low concentrations and degrades into harmless byproducts (like cyanuric acid). Compound **5**, as a derivative, inherits this “eco-friendly” pedigree. The data in Table 5 suggests that we have successfully engineered a molecule that retains the potent antimicrobial character of TCCA while potentially offering a more targeted or stable mode of action. Achieving complete inhibition at a concentration of just 100 µM (or 0.01%) is a highly promising result for a practical hatchery application.

Ultimately, the ability of compound **5** to achieve complete control at a low concentration, coupled with the environmentally benign nature of its chemical backbone, positions it as a highly attractive and scientifically validated candidate to replace harsher, traditional fungicides. This research paves the way for a future where fish hatcheries can be protected by chemistry that is not only effective but also in harmony with the aquatic environment.

Several plant extracts were evaluated to suppress the zoospores of *Saprolegnia* [36,37]. Bailey screened the effects of 25 chemicals representing seven classes of compounds for 15 and 60 min, on four species of aquatic fungi (*Saprolegniales*) pathogenic to fish. He found that the antifungal activity varied when each chemical was compared with that of malachite green, a reference compound [38].

### 3.5. Molecular Docking Studies

#### 3.5.1. Molecular Docking of Compound **5** into the Active Site of TS

The strong in silico prediction provides a compelling molecular basis for the potent cytotoxic effects observed for compound **5** against cancer cell lines, particularly human pancreatic (Mi-aPaCa2) cancer cells and human colon (HT29) cancer cells, where TS expression is often upregulated [26]. The data in Table 6 strongly suggest that the mechanism of cytotoxicity is, at least in part, mediated through the potent inhibition of Thymidylate Synthase. Analysis of the lowest-energy docking pose reveals that compound **5** fits snugly within the large, well-defined catalytic pocket of TS, establishing an extensive network of stabilizing interactions with key active site residues (Figure 3).

When comparing the binding mode of compound **5** to that of the reference inhibitors, the reasons for its superior binding energy become clear. While the reference inhibitor occupies the same general pocket and forms some of the same key interactions, its smaller, less complex structure limits its ability to engage the full range of available binding determinants. Generally, compound **5** is unequivocally the preferable docking score, the large, branched scaffold of compound **5** allows it to extend into and form interactions with multiple sub-pockets simultaneously, including the hydrophobic region. Compound **5** establishes a more extensive and diverse network of interactions. The critical π-π stacking with Phe117 and the intricate water-mediated hydrogen bonding network are unique features of its binding mode that are not observed for the simpler reference inhibitor. These additional interactions collectively contribute to the more favorable binding energy. The conformation and size of compound **5** demonstrate a higher degree of shape complementarity with the TS active site, maximizing favorable van der Waals contacts and minimizing steric clashes, which is reflected in its superior docking score.

#### 3.5.2. Molecular Docking Analysis into the CYP51 Active Site

A deeper breakdown of the energy components reveals further insights: although the reference inhibitor exhibits stronger raw interaction (E_Int) and hydrogen bond energies (E_H.B), compound **5** has a more favorable overall binding energy (BE), as it adopts a low-energy, stable conformation within the active site unlike the reference, which likely undergoes strain to fit, leading to higher energetic penalties [25]. Furthermore, the lower Root Mean Square Deviation (RMSD) for compound **5** (1.13 Å) indicates a more well-defined and stable binding pose compared to the reference (1.51 Å). The lowest-energy binding pose of compound **5** reveals an intricate and extensive network of interactions that anchor it firmly within the catalytic site of CYP51 (Figure 3). Predicted 3D binding mode of compound **5** within the active site of CYP51 (PDB ID: 5V5Z). The most critical interaction for CYP51 inhibition is the coordination of a ligand heteroatom to the central iron of the enzyme’s heme prosthetic group. The analysis shows that one of the triazine nitrogen atoms of compound **5** is perfectly positioned at a distance of ~2.2 Å from the heme iron, effectively blocking the enzyme’s catalytic function. This mode of action mimics that of established azole antifungals like fluconazole and voriconazole. The polar groups of compound **5** form a robust hydrogen bond network. The carbonyl oxygens and amine groups of the oxyethylamine side chains establish crucial hydrogen bonds with the side chains of Thr311, Gln479, and the highly conserved Arg381, providing significant enthalpic contributions to binding affinity. The large, lipophilic active site of CYP51 is well-occupied by the extensive scaffold of compound **5**. The side chains of the ligand nestle into a hydrophobic channel lined by residues such as Leu204, Ile304, Pro375, and Ile379, maximizing favorable van der Waals contacts and displacing water molecules from the active site. The aromatic triazine rings of compound **5** engage in favorable π-π stacking or T-shaped interactions with the phenolic rings of Tyr132 and Tyr158, which line the walls of the active site. Additionally, a potential π-sulfur interaction is observed between a triazine ring and the sulfur atom of Met374, further stabilizing the ligand’s orientation within the pocket [39]. Based on a holistic evaluation of the docking results, Compound **5** is unequivocally selected as the preferable inhibitor. The justification for this selection is multi-faceted. It possesses a more negative overall binding energy, indicating a thermodynamically more stable complex. Unlike the reference, which appears to rely on very strong but potentially strained interactions (as suggested by the high |E_Int|), compound **5** achieves its high affinity through a well-distributed network of moderate-to-strong interactions, including heme coordination, hydrogen bonding, and extensive hydrophobic contacts. This balanced “multi-pronged” binding strategy often leads to more robust inhibition and a lower energetic cost for binding. The large and flexible structure of compound **5** allows it to simultaneously occupy the heme-coordination site and the hydrophobic access channel, demonstrating a higher degree of shape complementarity with the entire active site compared to a potentially smaller reference inhibitor.

### 3.6. Quantum Chemical Analysis: Electronic Structure and Reactivity

A small energy gap is a hallmark of a chemically reactive and easily polarizable molecule, as it requires less energy to promote an electron to an excited state. This high reactivity is a fundamental prerequisite for potent biological activity, as it facilitates more effective orbital overlap and charge transfer with the biological target. Hardness (η) and Softness (σ), Following directly from the energy gap, compound **5** is the least “hard” (η = 0.80) and by far the “softest” (σ = 1.25) molecule in the series. According to Pearson’s Hard and Soft Acid-Base (HSAB) principle, soft molecules readily interact with soft biological sites. The high softness of compound **5** indicates that its electron cloud is easily deformed, allowing it to adapt its shape and electronic distribution to maximize favorable interactions within a binding pocket [24]. The most striking difference lies in the electrophilicity index (ω), which measures a molecule’s capacity to stabilize itself by accepting electronic charge. Compound **5** exhibits an exceptionally high electrophilicity index (ω = 13.34), an order of magnitude greater than its precursors. This quantifies it as a powerful electrophile (electron acceptor). This high ω value is the key quantum mechanical descriptor that rationalizes its potent cytotoxic and antifungal activities. It suggests a mechanism of action driven by its ability to accept electrons from crucial biological nucleophiles, thereby deactivating essential enzymes or damaging DNA. The quantum chemical analysis provides the “why” behind the molecular docking “how” generally the high electrophilicity (ω) and the localized LUMO of compound **5** explain its potent binding to Thymidylate Synthase (TS). The docking simulations showed that compound **5** forms key interactions with nucleophilic residues like Glu227. The DFT results confirm that compound **5** is electronically “primed” to accept charge from this glutamate residue, leading to the exceptionally stable complex and potent inhibition observed. Similarly, in the CYP51 active site, the primary mode of inhibition is coordination to the heme iron. The high electrophilicity of compound **5** makes its triazine nitrogen strong Lewis acid, facilitating its coordination to the electron-rich heme center. The high softness allows its electron cloud to deform and engage in the extensive hydrophobic and π-interactions with Tyr132, Met374, etc., as seen in the docking pose.

### 3.7. Protein Dynamics and Mechanism of Inhibition: A Normal Mode Analysis (NMA) Perspective

#### 3.7.1. NMA of the Compound **5**–1HZW Complex

The NMA of the smaller (TS) complex also provides critical insights (Figure 5a).

Deformability and B-factor analysis: The most striking feature for the TS complex is the excellent agreement between the NMA-predicted B-factors (red line) and the experimental crystallographic B-factors (black line). This high degree of correlation lends significant confidence to both the NMA model and, by extension, the stability and crystallographic relevance of the docked pose of compound 5. The deformability plot identifies key flexible regions, particularly a prominent loop around residues 150–180, which is known to be involved in the conformational changes required for catalysis.

Correlated motion and collective dynamics: The covariance matrix again shows correlated motions, and the cumulative variance plot indicates that the protein’s dynamics are dominated by a few low-frequency modes. This suggests that, like CYP51, TS relies on collective motions to perform its function.

#### 3.7.2. NMA of the Compound **5**-CYP51 (5V5Z) Complex

The covariance matrix (Figure 6d) shows large red blocks, indicating that entire domains of the protein move in a concerted, correlated fashion. This suggests a “breathing” or hinge-bending motion is essential for the enzyme’s function, likely to allow substrate entry and product release. The presence of blue regions indicates anti-correlated motions, characteristics of domains moving in opposition to each other. The cumulative variance plot (Figure 5e) demonstrates that the first 20 low-frequency modes account for over 90% of the total variance in the protein’s motion. The very small eigenvalue of the first mode (3.41) confirms that this dominant motion is a large-amplitude, collective movement of the entire protein structure. The NMA suggests that the catalytic function of CYP51 is dependent on the flexibility of its access channel loops and the collective “breathing” motion of its domains. Compound **5**, with its large, multi-pronged structure, acts as a highly effective “molecular plug.” It not only directly inhibits catalysis by coordinating with the heme iron (as seen in docking) but also physically occupies the vast active site cavity. By forming extensive interactions with the rigid core residues, it stabilizes the enzyme in a closed, inactive conformation. This prevents the essential large-scale conformational changes required for substrate access, effectively jamming the enzyme’s dynamic machinery. The docked pose is therefore highly stable and represents a functionally inhibited state of the enzyme (Figure 5f).

### 3.8. In Silico ADMET and Drug-likeness Prediction

The analysis reveals a clear profile for compound **5**. It’s very high molecular weight (996.81 g/mol) and topological polar surface area (TPSA > 500 Å^2^) are the primary drivers for its predicted low gastrointestinal absorption and inability to cross the blood-brain barrier. This can be advantageous for an anticancer or antifungal agent, as it may minimize central nervous system side effects. The compound is predicted to be a substrate for P-glycoprotein, which could contribute to resistance mechanisms in vivo. A highly favorable finding is that it is not an inhibitor of any major cytochrome P450 isoforms, suggesting a low potential for pharmacokinetic drug-drug interactions.

Concerning drug-likeness, compound **5** significantly violates the standard rules (Lipinski, Veber, etc.) due to its large, complex macrocyclic structure. This is visually confirmed by the bioavailability radar (Figure 7), where the pink area (compound’s properties) falls outside the optimal range (light pink) for all six parameters—LIPO (lipophilicity), SIZE, POLAR, INSOLU (insolubility), INSATU (insaturation), and FLEX (flexibility). This profile is typical for many potent, non-orally administered drugs, such as cyclosporine or vancomycin, which function effectively despite not conforming to the “Rule of 5.”

Critically, the analysis flagged zero PAINS (Pan-Assay Interference Compounds) or Brenk alerts, indicating that the observed biological activities are unlikely to be artifacts from promiscuous or reactive substructures. This strengthens the validity of our in vitro results. The synthetic accessibility score of 6.95 (on a scale from 1-very easy to 10-very difficult) accurately reflects the complex, multi-step assembly of this macromolecule.

In conclusion, the in silico ADMET profile positions compound **5** as a promising candidate for non-oral applications (e.g., intravenous for anticancer use or topical/aquaculture for antifungal use). Its large, polar structure ensures targeted action with a low risk of CNS side effects or CYP-mediated interactions, while the absence of toxicity alerts underscores its potential as a specific, rather than promiscuous, bioactive agent.

Figure 7 bioavailability radar for compound **5**. The pink area represents the physicochemical space of the compound, while the light pink zone indicates the optimal range for oral bioavailability. The compound’s profile falls outside the ideal range for all parameters (LIPO: lipophilicity, SIZE: molecular weight, POLAR: polarity, INSOLU: insolubility, INSATU: insaturation, FLEX: flexibility), consistent with its high molecular weight and polarity, and predicting low oral bioavailability.

## 4. Materials and Methods

### 4.1. Chemistry

IR spectra were taken as KBr disk on IR model JASCO FT/IR 410. NMR were recorded on Bruker 400 MHz Spectrometer (Bruker-BioSpin, Faellanden, Switzerland) in the solvent deuterated Dimethyl sulfoxide-d6 (DMSO-d6). Chemical shifts were reported in (*δ*) ppm; TMS was used as internal reference; signals were quoted as (*s*: singlet, *d*: doublet, *t*: triplet, *b s*: broad singlet, *q*: quartet, *m*: multiplet). NMR were recorded in NMR laboratory, Homs University, Homs, Syria. Mass spectra were recorded using Shimadzu Gc-17A, Gc-Ms-QP5050A spectrometer in Damascus, Syria and using Acquisition SW: 6200 series TOF/6500 series, Version Q-TOF B.08.00 (B8058.0) in Eastern Anatolia High Technology Application and Research Center (DAYTAM), Ataturk University, Erzurum, Turkey. TLC was carried out on 0.25 mm layers using silica gel-G coated Al-plates containing a fluorescent indicator; spots were detected under UV light (254 nm) and Iodine. Chemicals used in this study were obtained from Merck (Merck, Darmstadt, Germany).

EA (10 mmol), solved in (20 mL) acetonitrile, was added to a solution of TCCA (30 mmol) solved in (30 mL) acetonitrile. The reaction mixture was stirred at 80 °C for 4 h. The TLC tracked reaction progress. From the filtrate, a light-yellow powder was obtained, which was recrystallized with (Ac-etone: Ethanol: Water 2:5:1). A shiny light-yellow powder appeared with 0.32 g 11.32% yield. According to mass spectrum, it was observed that the shiny light-yellow powder was a mixture of compounds **3** and **4**. It was not possible to isolate compounds **3** and **4** by chromatographic methods, because these compounds gave one spot in different mobile phases. Compound **3** is: Bis(^1^N,^3^N)(Oxyethylamine)-^5^N chloroisocyanuric acid and compound **4** is: Tri(^1^N,^3^N,^5^N) (Oxyethylamine) isocyanuric acid. The precipitate formed after cooling was filtered and washed with acetone. The precipitate resulting from the basic reaction was recrystallized with acetone:ethanol:water (1:3:1) to yield pure acyclic nucleoside 5. White, lustrous, acicular crystals, 2.37 g, yield 84.19%, positive density 245 °C, TLC (Tolu:MeOH 2:1) Rf = 0.2. This compound dissolved in dimethyl sulfoxide (DMSO). Qualitative chlorine test gave negative results, confirming the absence of chlorine atoms in compound **5**: Tri (^1^N,^3^N,^5^N) (Oxyethyl) [tri (^1′^N, ^1′′^N, ^1′′′^N) (^3′^N, ^5′^N, ^3′′^N, ^5′′^N, ^3′′′^N, ^5′′′^N) hexa (Oxyethylamine) isocyanuric acid] isocyanuric acid.

### 4.2. Anticancer Studies

In this study, the MTT assay, a widely used method for evaluating anticancer activity and cell viability, was employed to assess the cytotoxic effects of the tested compound. The basic principle of MTT analysis is based on the reduction of MTT to formazan crystals by means of living cells and is applied to determine mitochondrial activity. Cell culture analysis was performed in HT-29 [American Type Culture Collection (ATCC), Manassas, VA, USA; cat no: HT-29 HTB-38], OVCAR3 (ATCC, cat. no. HTB-165), MIAPACA2 (ATCC, cat. no. CRL-1420), and T98G cell (ATCC, cat. no. CRL-1690) lines as follows [40,41]. Dulbecco’s modified Eagle’s medium (DMEM) was used as the medium. The nutrient medium was added to the medium containing 10% Fetal Bovine Serum (FBS, Sigma–Aldirch, Saint Louis, MO, USA), 1% L-Glutamine and 1% Penicillin streptomycin (Sigma–Aldirch, USA). HT-29, OVCAR3, MIAPACA2, and T98G cell lines were propagated in a 5% CO_2_, 37 °C incubator. After cell counting, 1 × 10^4^ cells were seeded into each well. DMSO at concentration (1%) was used as a solvent to prepare the dilutions of compound **5**. After incubation, different concentrations of the sample (62.75, 125.5, 251, 502, and 1004 µM ) were prepared and passed through a 0.22 µM (PVDF, Merck Millipore, Burlington, MA, USA) filter and 10 µL were seeded into 96 well plates. Then, MTT, which is a cell viability test, was studied at 24, 48 and 72 h. After MTT was added, absorbance was measured in a microplate reader (Thermo MultiskanGo, Thermo Fisher Scientific, Waltham, MA, USA, 570 nm) at the end of three hours of incubation [41].

### 4.3. Antioxidant Studies

#### 4.3.1. DPPH Method

The DPPH method is based on the reduction of the stable DPPH radical (0.1 mM in ethanol) by antioxidants, leading to a decrease in absorbance at 517 nm. Compound **5** and ascorbic acid (AscA, positive control) were prepared in distilled water at concentrations ranging from 1004 to 62.75 µM. In a 96-well plate, 20 µL of sample or AscA was mixed with 180 µL of DPPH solution and incubated in the dark for 30 min. Absorbance was recorded using a microplate reader (Thermo MultiskanGo, USA, 517 nm). Each sample was tested in triplicate. The percentage of DPPH scavenging was calculated based on the absorbance of control and samples. DPPH radical scavenging activity is calculated by the following equation:

DPPH Inhibition Rate (%) = [(A_control – A_sample)/A_control] × 100

A_control: Absorbance of the control.

A_sample: Absorbance of the sample or AscA.

#### 4.3.2. CUPRAC Method

The CUPRAC method evaluates antioxidant capacity based on the reduction of Cu(II) to Cu(I) by antioxidants using the bis(neocuproine)-copper(II) chloride reagent [42]. In a 96-well plate, 60 µL each of Cu(II) chloride, neocuproine (in ethanol), and ammonium acetate buffer (pH 7.0) were combined with 20 µL of sample or AscA. After incubation in the dark at room temperature for 30 min, absorbance was measured at 450 nm.

### 4.4. Antifungal Studies

Zoospore suspension of *Saprolegnia parasitica* was prepared as previously described [37]. In brief, the following procedures were followed: subculturing the pathogenic isolate on potato dextrose agar for three days; agar discs from the edge of actively growing *Saprolegnia parasitica* were cut and immersed in sterilized fish tank water; flasks were incubated at 18 ± 2 °C for 18 h and the agar discs were discarded. Following harvesting, the zoospores of *Saprolegnia parasitica* were adjusted to 30% absorbance at wavelength (λ) 580 nm using a spectrophotometer, corresponding to approximately 1.2 × 10^3^ zoospores/mL. *Saprolegnia parasitica* used in this study was kindly supplied and identified by the Fish Research Department, Faculty of Veterinary Medicine, Cairo University, Egypt.

Compound **5** was dissolved in DMSO and diluted with sterile water to obtain final test concentrations of 20, 40, 60, and 100 µM. Aliquots of the zoospore suspension (1 mL) were treated with the compound solutions for 3, 5, and 10 min of exposure at room temperature.

Two controls were included: sterile water as the negative control, and formalin (0.1% *v*/*v*) as the positive control. Each treatment was performed in triplicate, and the results are expressed as mean ± SD.

### 4.5. Molecular Docking Studies

#### 4.5.1. Computational Methods

All quantum chemical calculations were performed using the Gaussian 16, Revision C.01 software package [43]. The initial three-dimensional structures of compounds were constructed using the Gauss View 6.0 graphical interface. Full geometry optimization of each compound was carried out in the gas phase without symmetry constraints using Density Functional Theory (DFT). The calculations employed the Becke, 3-parameter, Lee-Yang-Parr (B3LYP) hybrid functional [44], which has been extensively validated for its accuracy in describing the electronic properties of organic molecules. The 6-311G++(d,p) basis set was used for all atoms, which includes diffuse functions (++) to accurately describe anions and lone pairs, as well as polarization functions (d,p) to account for the non-uniform distribution of electron density in bonding environments. The optimization was considered complete when the forces on the atoms were less than 0.00045 Ha/Bohr and the displacement for the next optimization step was less than 0.0018 Bohr. To confirm that the optimized geometries corresponded to true local minima on the potential energy surface, harmonic vibrational frequency calculations were performed at the same B3LYP/6-311G++(d,p) level of theory. The absence of any imaginary frequencies for all optimized structures verified their stability. The energies of the EHOMO) and the (ELUMO) were obtained directly from the output of the optimized structures. These energies were then used to calculate a series of global reactivity descriptors based on conceptual DFT. The ionization potential (IP) and electron affinity (EA) were approximated within the framework of Koopmans’ theorem (IP ≈ −EHOMO; EA ≈ −E>LUMO). The following key reactivity indices were calculated using the standard equations: (ΔƐ) = ELUMO—EHOMO, (η): η = (ELUMO—EHOMO)/2 (σ): = 1/η,μ = (EHOMO + ELUMO)/2 ≈ −χ (ω) μ^2^/(2η). The three-dimensional graphical representations of the HOMO and LUMO molecular orbital surfaces were generated using the GaussView 6.0 software package.

#### 4.5.2. Molecular Docking Methodology

To investigate the binding interactions and predict the affinity of compound **5** and the reference inhibitors with their respective biological targets, molecular docking simulations were performed using the AutoDock Vina 1.1.2 program [45]. The three-dimensional crystal structures of the target enzymes, (PDB ID: 1HZW) and (CYP51, PDB ID: 5V5Z), were retrieved from the RCSB Protein Data Bank. The protein structures were prepared for docking using the AutoDockTools (ADT) 1.5.6 suite. Preparation involved the removal of all water molecules, co-crystallized ligands, and any non-essential ions from the PDB files. Subsequently, polar hydrogen atoms were added to the protein structures, and Gasteiger partial charges were computed and assigned to all atoms. The prepared protein files were saved in the PDBQT format, which includes atomic coordinates, charge information, and atom type specifications required by AutoDock Vina. The grid box was defined for each protein to encompass the entire active site and provide sufficient space for the ligand to translate and rotate freely. The center of the grid box was determined based on the geometric center of the co-crystallized ligand in the original PDB file, ensuring the search was focused on the known binding cavity. For both 1HZW and 5V5Z, the grid dimensions were set to 60 × 60 × 60 Å with a grid point spacing of 1.0 Å. The docking calculations were executed using AutoDock Vina with the exhaustiveness parameter set to 10, which controls the thoroughness of the conformational search. Vina’s scoring function, which approximates the binding energy in kcal/mol, was used to rank the resulting poses. For each docking run, a total of nine distinct binding conformations were generated and ranked. The binding pose with the most negative (i.e., most favorable) binding energy was selected for detailed interaction analysis. The protein-ligand interaction patterns, including hydrogen bonds, hydrophobic interactions, and π-stacking, were analyzed and visualized using the Dassault Systèmes BIOVIA Discovery Studio Visualizer 2021 client [46]. Two-dimensional and three-dimensional representations of the binding modes were generated to illustrate the key stabilizing interactions within the active site of each enzyme.

#### 4.5.3. Normal Mode Analysis (NMA) and Protein Dynamics

To investigate the intrinsic, low-frequency collective motions inherent to the pdb, Normal Mode Analysis (NMA) was performed. This analysis was conducted using the ProDy computational library (v2.1) [47] integrated within a Python 3.9 environment. The analysis was based on the high-resolution crystal structure. The Anisotropic Network Model (ANM) was employed for the NMA calculations [48]. The ANM is a coarse-grained elastic network model (ENM) that has been demonstrated to effectively capture the large-scale, functional dynamics of proteins. In this model, the protein is represented by a network of nodes, with each Cα atom serving as a node. Nodes within a defined cutoff distance are connected by harmonic springs, all with a uniform force constant. A distance cutoff of 15.0 Å was used to define the interactions between Cα atoms. The Hessian matrix, which describes the second derivatives of the potential energy with respect to atomic coordinates, was constructed for the Cα network. Diagonalization of this matrix yielded a set of 3*N*-6 non-trivial eigenvectors and their corresponding eigenvalues, where *N* is the number of Cα atoms. The eigenvectors represent the normal modes, which are the collective directions of tomic motion, while the eigenvalues represent the frequencies (and thus the energy) associated with each mode. The first 20 low-frequency, non-trivial modes were selected for further analysis, as these are known to correspond to the most functionally relevant, large-amplitude conformational changes in the protein. All visualizations of the NMA results, including fluctuation plots, deformability profiles, and cross-correlation maps, were generated using the. Matplotlib (v3.9.2) with the native plotting functionalities of ProDy.

### 4.6. Statistical Analysis

In the MTT method used in the anticancer study of compounds, the groups included in the study were run in six repetitions and the mean of the data was shown in graphs with their standard deviations after the study. The results of the graphs related to the study were obtained using the “Nonlinear Regression-[Iinhibitor] vs. Response (Three Parameters)” test in the GraphPad Prism 8 program. The results of the relevant graphs belonging to the antioxidant studies were obtained using the same method. The results of antifungal evaluation were recorded as mean ± SD from three replicates for each treatment.

## 5. Conclusions

This research culminates in the successful design, synthesis, and evaluation of compound **5**, a novel acyclic heterocyclic macromolecule with a remarkable dual-action profile. Through a simple, high-yield, one-pot reaction, we constructed a complex molecular architecture from readily available starting materials, TCCA and ethanolamine. Comprehensive spectroscopic analysis, including high-resolution mass spectrometry and ^2^D NMR, provided an unequivocal confirmation of its unique, tetrameric structure. Biologically, compound **5** emerged as a promising therapeutic agent with a distinct and selective personality. It demonstrated significant and dose-dependent cytotoxic activity against pancreatic (MiaPaCa2) cancer cells, while this cytotoxic activity was less effective against human colon (HT29) and ovarian (OVCAR3) cancer cells cancers. This selective cytotoxicity was strongly supported by our in silico studies, which revealed a superior binding affinity for key oncogenic enzymes like Thymidylate Synthase. Furthermore, compound **5** proved to be a potent antifungal agent, achieving the complete inhibition of the devastating water mould *Saprolegnia* at low concentrations, positioning it as a viable and eco-friendly alternative to traditional chemical treatments. In conclusion, this work introduces compound **5** not merely as a new molecule, but as a validated lead candidate with multifaceted potential. It stands at the intersection of medicinal chemistry and green technology, offering a clear path forward for the development of targeted anticancer therapies and safer, more sustainable solutions for aquaculture.

## Figures and Tables

**Table 1:** Physical data of compounds **3**, **4**, and **5**.

Compound	Molecular Weight (g/mol)	Molecular Formula	Yield (%)	M.P. (°C)	Rf (Tolu:MeOH)
**3**	281	C_7_H_12_N_5_O_5_Cl	11.32	Dec. 252–258	0.28 (1:1)
**4**	306	C_9_H_18_N_6_O_6_	11.32	Dec. 252–258	0.28 (1:1)
**5**	996	C_30_H_48_N_18_O_21_	84.19	Dec. 245	0.20 (2:1)

Rf: TLC Retention Factor; M.P.: Melting point; Dec.: Decomposes.

**Table 2:** Characteristic fragments of the mass spectrum (CI/Ms) for compound **3**.

*m*/*z*	Relative Abundance (%)	*m*/*z*	Relative Abundance (%)	*m*/*z*	Relative Abundance (%)
71	28.68	132	2.96	281	4.96
70	27.96	113	13.64	279	6.03
69	1.87	112	6.44	253	1.12
57	46.83	105	4.81	178	1.58
56	10.00	104	8.60	168	2.78
55	24.56	84	4.74	167	35.61
43	26.37	83	11.85	163	1.66
42	6.12	76	5.68	150	9.92
41	29.76	72	9.50	149	100.00

**Table 3:** Characteristic fragments of the mass spectrum (CI/Ms) for compound **4**.

*m*/*z*	Relative Abundance (%)	*m*/*z*	Relative Abundance (%)	*m*/*z*	Relative Abundance (%)
70	14.26	233	0.26	389	0.32
63	1.11	209	0.23	379	0.50
62	2.69	196	0.51	370	0.50
48	3.73	184	0.53	365	0.57
44	75.69	175	0.43	363	0.46
43	80.81	130	2.76	352	0.34
42	16.99	129	100.00	324	0.78
41	2.87	87	1.47	310	0.08
40	7.93	86	16.64	308	0.17

**Table 4:** Characteristic fragments of the mass spectrum (Q-TOF/MS) for compound **5**.

*m*/*z*	Relative Abundance (%)	*m*/*z*	Relative Abundance (%)	*m*/*z*	Relative Abundance (%)
62	87.00	284	1.44	453	6.11
63	1.83	286	1.48	491	1.82
64	54.13	391	7.59	512	5.14
79	77.10	392	1.78	513	1.62
80	1.83	413	100.00	540	2.40
81	35.62	414	23.68	803	25.86
101	87.69	415	3.22	804	12.85
102	3.38	429	3.62	805	3.29
117	1.93	452	23.98	983.7	1.34
140	3.81			984.7	0.82
150	2.05			997.69	1.43
157	17.13			998.69	0.88

**Table 5:** Eco-friendly potential antifungal activity of compound **5** (TCCA derivative) for different exposure times against *Saprolegnia parasitica* in controlling diseases of fish hatcheries. * & **.

Exposure Time (Minutes)	3	5	10
Dose (µM)	Inhibition (%)	Inhibition (%)	Inhibition (%)
20	8.42 ± 2.11	12.42 ± 3.51	18.42 ± 2.11
40	12.71 ± 3.23	18.21 ± 4.25	32.75 ± 3.23
60	27.02 ± 4.51	41.20 ± 7.43	65.22 ± 5.61
100	53.4 ± 5.22	88.5 ± 7.3	100
Control 1 (Negative control; water)	0.0	0.0	0.0
Control 2 (Positive control: formaline)	9.3 ± 2.6	11.3 ± 2.1	14.2 ± 2.6

* The initial number of spores was adjusted to 1.2 × 10^3^/mL. ** Data were recorded as mean ± SD from three replicates for each treatment.

**Table 6:** Binding energies of compound **5** and reference inhibitors (1YN, Raltitrexed, dUMP and Gol.) docked into the active sites of Thymidylate Synthase (PDB: 1HZW) and CYP51 (PDB: 5V5Z).

	BE	rmsd	E_Int	E_H.B
5V5Z				
5	−9.874	1.125	−45.263	−30.698
1YN	−9.551	1.514	−107.02	−38.643
1HZW				
5	−5.454	1.534	−26.2266	−2.1429
Raltitrexed	−4.153	1.689	−13.209	−4.347
dUMP	−3.984	1.159	−13.314	−3.390

**Table 7:** Calculated quantum chemical parameters (global reactivity indices eV.) for compounds **3**–**5**.

Parameter	Compound 3	Compound 4	Compound 5
EHOMO	−6.91	−6.62	−5.42
ELUMO	−1.88	−1.65	−3.82
Energy Gap (ΔƐ)	5.03	4.97	1.60
Hardness (η)	2.52	2.49	0.80
Softness (σ)	0.397	0.402	1.25
Electronegativity (χ)	4.40	4.14	4.62
Electrophilicity (ω)	3.84	3.44	13.34

**Table 8:** In silico ADMET and drug-Likeness properties of compound **5** predicted by SwissADME.

Property/Predictor	Compound 5	Interpretation
Physicochemical Properties		
Molecular Weight (g/mol)	996.81	High
Num. H-bond acceptors	27	High
Num. H-bond donors	6	Moderate
Molar Refractivity	221.41	High
TPSA (Å^2^)	503.19	Very High
Pharmacokinetics		
GI absorption	Low	Poor oral absorption expected
BBB permeant	No	Unlikely to cross BBB
P-glycoprotein substrate	Yes	Subject to efflux
CYP Inhibitor (1A2, 2C19, 2C9, 2D6, 3A4)	No	Low risk of CYP-mediated drug interactions
Skin Permeability (log Kp, cm/s)	−18.34	Very low (unlikely to penetrate skin)
Drug-likeness		
Lipinski (Rule of 5)	3 violations (MW, HBA, Log P)	Not drug-like by strict oral standards
Ghose	4 violations	
Veber	2 violations	(Rot. bonds, TPSA)
Egan	1 violation	(TPSA)
Muegge	6 violations	
Bioavailability Score	0.17	Low
Medicinal Chemistry		
PAINS alerts	0	No pan-assay interference substructures
Brenk alerts	0	No problematic functional groups
Leadlikeness	2 violations	Not lead-like (MW, Rot. bonds)
Synthetic Accessibility	6.95	Very complex to synthesize

## Data Availability

The original contributions presented in this study are included in the article/Supplementary Material. Further inquiries can be directed to the corresponding author(s).

## Supplementary Materials

The following supporting information can be downloaded at: https://www.mdpi.com/article/10.3390/ph18101533/s1. Figure S1: CI/Ms spectrum of compound **3** and **4**; Figure S2: 1H NMR spectra for compound **5** in DMSO-d6 and in DMSO-d6 + D2O; Figure S3: 13C NMR and DEPT-135 spectra for compound **5** in DMSO-d6; Figure S4: 1H-1H COSY and HSQC spectra for compound **5** in DMSO-d6; Figure S5: LC/ Q-TOF/MS spectrum of compound **5**; Table S1: Cell viability rates of compound **5** in HT29, Miapaca 2, OVCAR3 and T98G cells; Table S2: DPPH and CUPRAC antioxidant activity of compound **5** and AscA.
