# Supplementary material for: Synthesis, Spectroscopic Characterization, and Biological Evaluation of a Novel Acyclic Heterocyclic Compound: Anticancer, Antioxidant, Antifungal, and Molecular Docking Studies
Source: Pharmaceuticals (Basel). 2025 Oct 12;18(10):1533. doi: 10.3390/ph18101533 (PMC12567457; doi:10.3390/ph18101533)
Supplement: Supplementary file 1 [file pharmaceuticals-18-01533-s001.zip › pharmaceuticals-3863748-supplementary.pdf]

Synthesis, Spectroscopic Characterization, and Biological Evaluation of a Novel Acyclic Heterocyclic Compound: Anticancer, Antioxidant, Antifungal, and Molecular Docking Studies

Mohammad Alhilal[1], Suzan Alhilal[2*], Ilhan Sabancilar[3], Sobhi M. Gomha[4*], Ahmed A. Elhenawy[5,6], Salama A. Ouf[7]

[1]Department of Nursing, Faculty of Health Sciences, Mardin Artuklu University, Mardin, Turkey; mohammadalhilal@artuklu.edu.tr

[2]Department of Medical Services and Techniques, Vocational School of Health Services, Mardin Artuklu University, Mardin, Turkey

[3]Department of Medical Services and Techniques, Vocational School of Health Services, Bitlis Eren University, Bitlis, Turkey; isabancilar@beu.edu.tr

[4]Department of Chemistry, Faculty of Science, Islamic University of Madinah, Madinah, Saudi Arabia

[5]Chemistry Department, Faculty of Science, Al-Baha University, Al-Baha, Saudi Arabia; elhenawy_sci@hotmail.com

[6]Chemistry Department, Faculty of Science, Al-Azhar University, Cairo, Egypt

[7]Botany & Microbiology Department, Faculty of Science, Cairo University, Giza, Egypt; saoufeg@yahoo.com

***Correspondence:** suzanalhilal@artuklu.edu.tr (S.A.) smgomha@iu.edu.sa (S.M.G.)

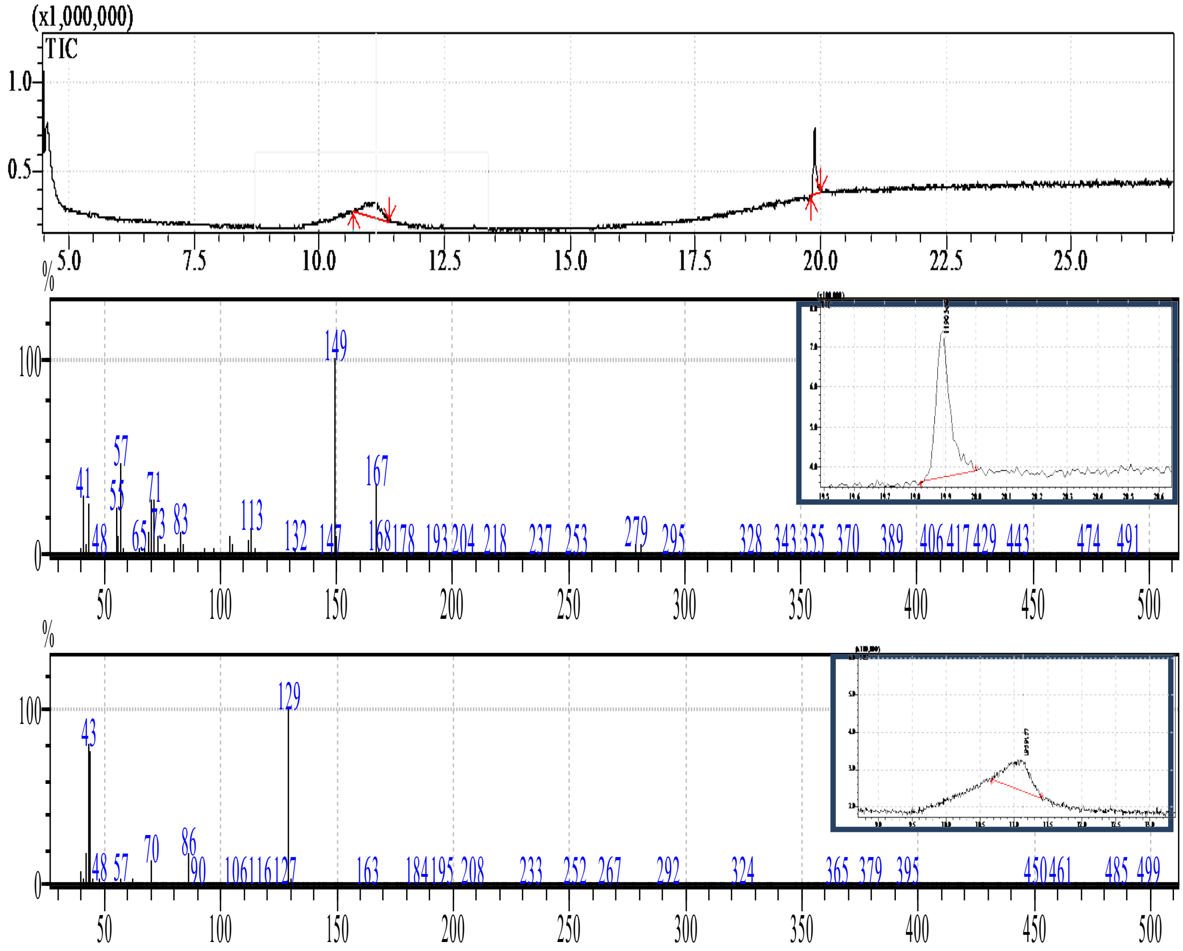

Figure S1: CI/Ms spectrum of compound 3 and 4.

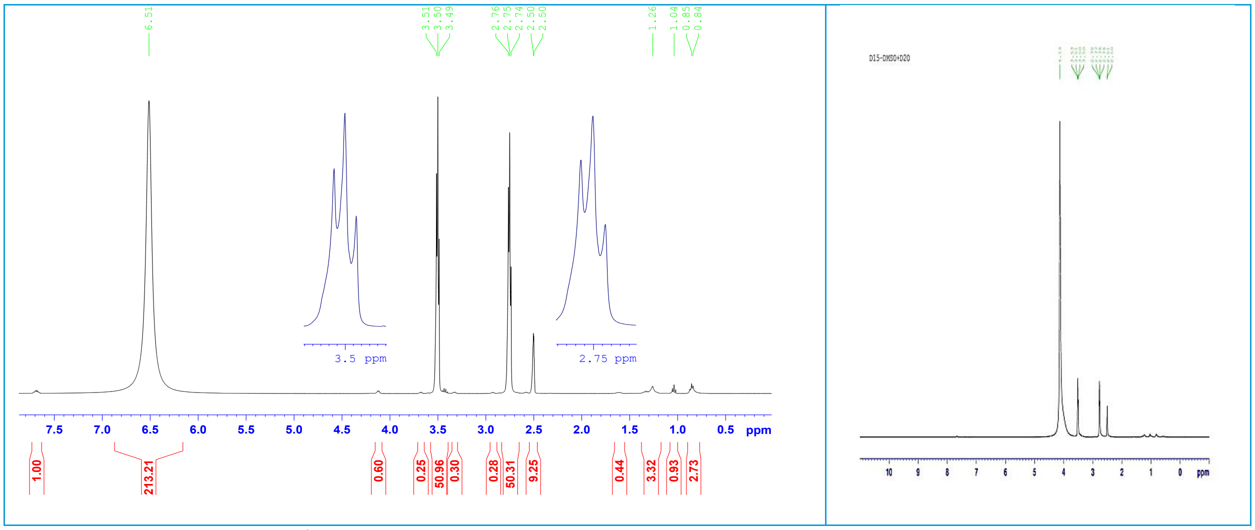

Figure S2: ^{1}H NMR spectra for compound 5 in DMSO-d6 and in DMSO-d6 + D_2O.

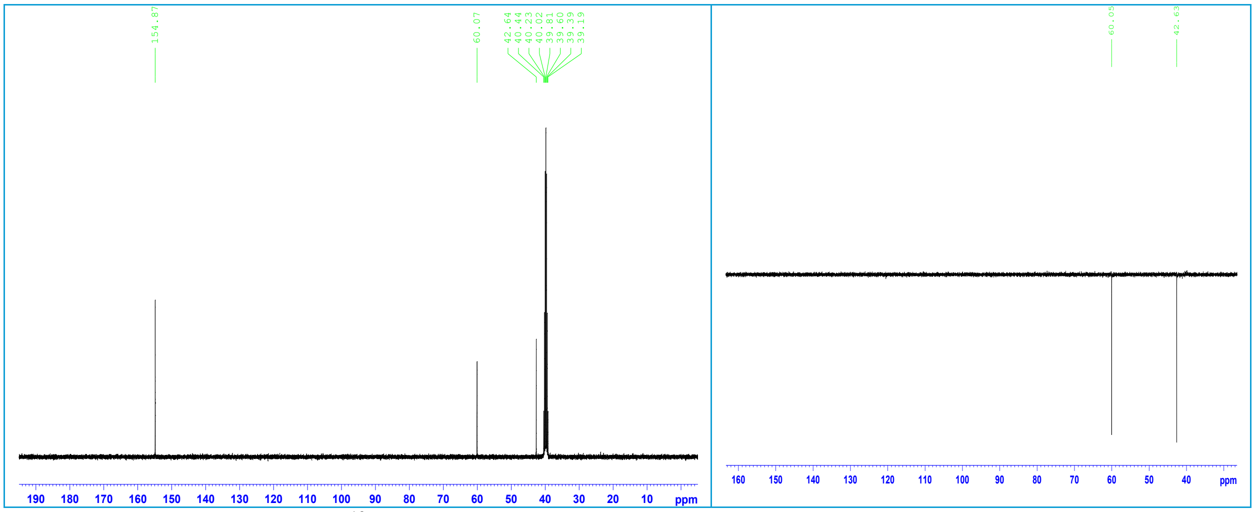

Figure S3: ^{13}C NMR and DEPT-135 spectra for compound 5 in DMSO-d6.

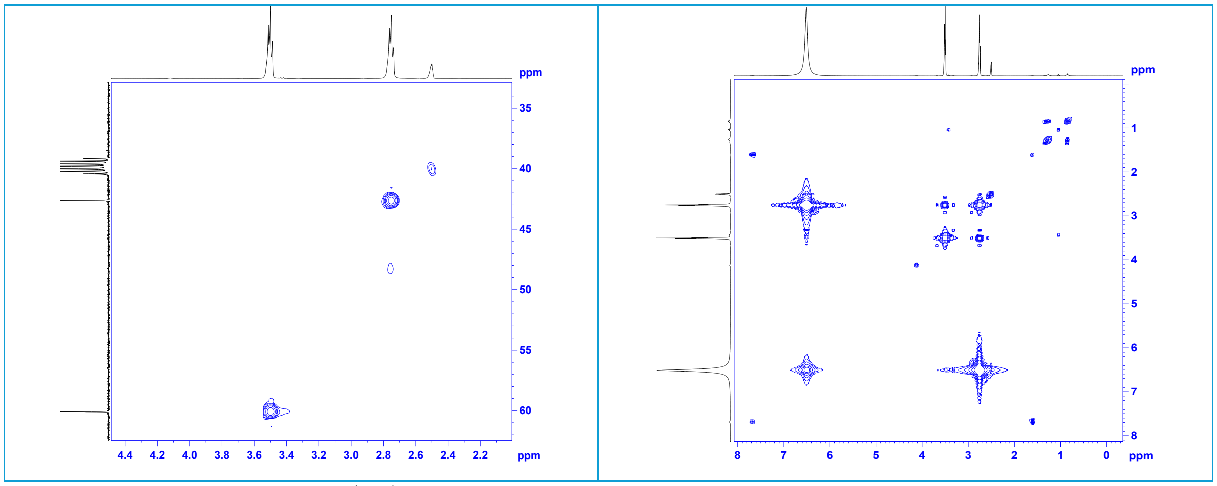

Figure S4: ^{1}H-^{1}H COSY and HSQC spectra for compound 5 in DMSO-d6.

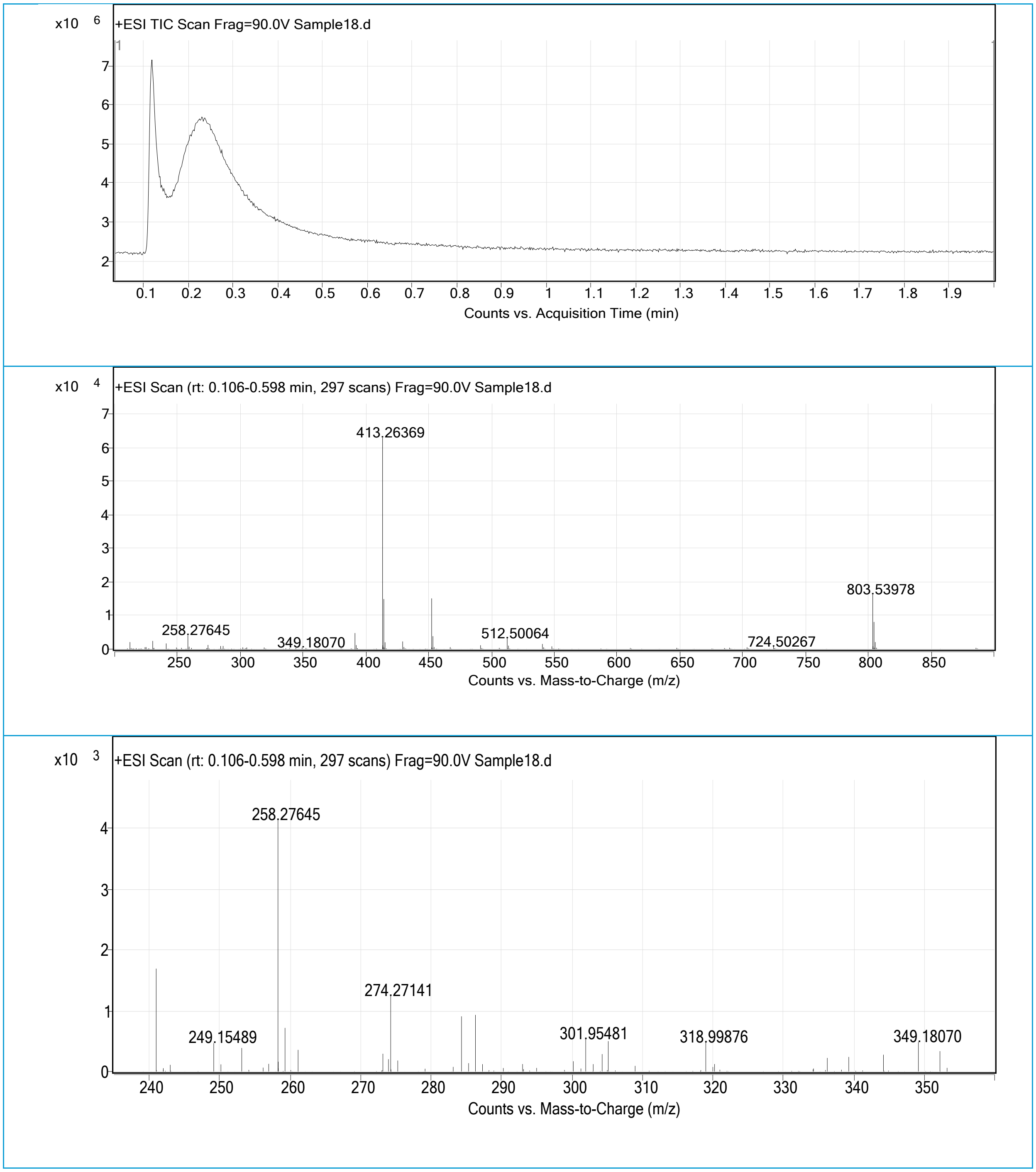

Figure S5: LC/ Q-TOF/MS spectrum of compound **5**.

Table S1: Cell viability rates of compound 5 in HT29, Miapaca 2, OVCAR3 and T98G cells

Cell Line	Dose (µM)	Cell Viability (Control) (Mean±SD)		
		24h	48h	72h
HT29	Control	100±0	100±0	100±0
	62.75	96.5±1.8	92.1±4.3	94.4±4.3
	125.5	92.5±3.4	87.1±2.7	92.6±3.9
	251	89.4±4.3	84.2±4.1	89.4±5.8
	502	88.9±4.4	83±3.5	85.2±5.3
	1004	86.1±1.7	82.3±3.7	79.7±5.1
	IC_{50}<br>R^2	149.89<br>0.7280	74.82<br>0.7972	393.9<br>0.7077
MiaPaCa2	Control	100±0	100±0	100±0
	62.75	95.8±2.3	94.9±5.9	95.9±1.9
	125.5	87.5±2.5	84.8±3.5	91.8±2.8
	251	83.1±2.6	80.8±2.2	87.1±3.1
	502	79.6±2.8	71.4±5.8	77.3±2.1
	1004	74.1±4.5	69.3±5	71±1.4
	IC_{50}<br>R^2	260.2<br>0.7505	247.7<br>0.7803	304.3<br>0.8787
OVCAR-3	Control	100±0	100±0	100±0
	62.75	92±3.3	96.3±2.3	98.9±0.9
	125.5	88.6±4.6	91.4±2	98.7±2
	251	85.7±5.4	87.9±3.7	95.9±4.2
	502	84.5±5.4	85.5±3.1	94.4±3.3
	1004	82.3±4.8	82.2±3.6	88.9±6
	IC_{50}<br>R^2	84.5<br>0.6835	204.7<br>0.8467	4535<br>0.5833
T98G	Control	100±0	100±0	100±0
	62.75	96±3.2	96±2.9	95.9±2.4
	125.5	92±4.3	89.7±6.2	90.6±4.7
	251	87.5±3.9	85.5±6	86.3±3.2
	502	85±5.7	81.5±3.7	82.5±1.1
	1004	80.5±4.9	77.2±5	76.9±4.2
	IC_{50}<br>R^2	261.2<br>0.7505	248.7<br>0.7803	305.5<br>0.8787

Three independent experiments were performed in triplicate. Data are given as mean ± standard deviation (SD).

Table S2: DPPH and CUPRAC antioxidant activity of compound 5 and AscA

	DPPH		CUPRAC	
Dose (μM)	DPPH• Inhibition Rate (%) (Mean±SD)		Absorbance $_{450nm}$ (Mean±SD)	
	AscA	Compound 5	AscA	Compound 5
1004	67.13±4.51	22.02±0.93	1.768±0.176	0.135±0.005
502	70.55±6.1	27.68±1.95	1.22±0.017	0.219±0.006
251	62.36±0.38	17.3±2.04	0.623±0.071	0.152±0.009
125,5	66.77±4.82	17.09±2.25	0.578±0.02	0.224±0.006
62.75	38.46±2.76	6.63±2.97	0.227±0.052	0.164±0.01
0	0	0	0.171±0.007	0.171±0.007
IC_{50}<br>R^2	38.5<br>0.9067	115.6<br>0.8195	1527<br>0.9713	IC50>high<br>0.1330

Three independent experiments were performed in triplicate. Data are given as mean ± standard deviation (SD).
